# Can’t help processing numbers with text: Eye-tracking evidence for simultaneous instead of sequential processing of text and numbers in arithmetic word problems

**DOI:** 10.1007/s00426-024-02069-x

**Published:** 2025-01-20

**Authors:** Lilly Roth, Hans-Christoph Nuerk, Felix Cramer, Gabriella Daroczy

**Affiliations:** 1 Department of Psychology, University of Tübingen, Tübingen, Germany; 2 LEAD Graduate School & Research Network, University of Tübingen, Tübingen, Germany; 3 German Center for Mental Health (DZPG), Mannheim, Germany

## Abstract

Solving arithmetic word problems requires individuals to create a correct mental representation, and this involves both text processing and number processing. The latter comprises understanding the semantic meaning of numbers (i.e., their magnitudes) and potentially executing the appropriate mathematical operation. However, it is not yet clear whether number processing occurs after text processing or both take place simultaneously. We hypothesize that number processing occurs early and simultaneously with other problem-solving processes such as text processing. To test this hypothesis, we created non-solvable word problems that do not require any number processing and we manipulated the calculation difficulty using carry/borrow vs. non-carry/non-borrow within addition and subtraction problems. According to a strictly sequential model, this manipulation should not matter, because when problems are non-solvable, no calculation is required. In contrast, according to an interactive model, attention to numbers would be higher when word problems require a carry/borrow compared to a non-carry/non-borrow operation. Eye-tracking was used to measure attention to numbers and text in 63 adults, operationalized by static (duration and count of fixations and regressions) and dynamic measures (count of transitions). An interaction between difficulty and operation was found for all static and dynamic eye-tracking variables as well as for response times and error rates. The observed number processing in non-solvable word problems, which indicates that it occurs simultaneously with text processing, is inconsistent with strictly sequential models.

**Supplementary Information:**

The online version contains supplementary material available at 10.1007/s00426-024-02069-x.

## Introduction

Word problem solving requires both text processing and number processing. That is, the text must be read and understood, and the numbers must be identified, semantically understood in terms of their magnitude, and potentially used for calculations. Text processing and number processing might take place at distinct levels, which are strictly sequential according to some models (i.e., first text processing, then number processing), while other models assume an interaction between text processing and number processing.

Surprisingly, far more studies have investigated text processing and linguistic features than number processing and numeric features in word problems (see Jaffe & Bolger, 2023, for a review). Nevertheless, numbers are most often relevant for solving word problems. Interestingly, there is increasing evidence for number processing even when numbers are not required for the solution. In this work, number processing refers to identifying numbers, semantically understanding their magnitude, and potentially also calculating in terms of performing mathematical operations. For example, in a study by Reusser and Stebler (1997), most people solved the following word problem incorrectly: “There are 26 sheep and 10 goats on a ship. How old is the captain?” performing an addition or subtraction operation with the numbers. People tend to use the numbers even when no calculation is required. One explanation for this is that people do not connect word problems to real-world scenarios and expect that every word problem is solvable (e.g., Jiménez & Verschaffel, 2014; Reusser & Stebler, 1997). However, this explanation suggests that number processing reflects a mistake happening when text processing is erroneous. With such assumptions, attempted solutions for non-solvable word problems could still be consistent with a sequential model. It would be highly interesting to find out whether problem solvers also process numbers when they correctly respond to non-solvable problems (i.e., in cases where they realize the non-solvable nature of the word problem).

The challenge in exploring this question is that standard behavioral measures like overall response times (RT) or error rates (ER) often fail to detect whether early number processing occurs while the text is still being processed (although advanced difficulty manipulation might help, see below). Therefore, in this study, we aim to investigate number processing (including early steps, such as understanding the semantic meaning of the numbers’ magnitudes, and potentially also the execution of a mathematical operation) in both solvable and non-solvable word problems with eye-tracking while manipulating number difficulty.

### Number processing in word problems

Solving word problems requires solvers to create a correct mental representation (sometimes also referred to as ‘mental model’) of the word problems. To our understanding, a mental representation is a mental framework that individuals build during problem-solving. It combines all key information—like details in the text, the situation, potentially required mathematical operations, and involved numbers—in a single, organized structure. Mental representations are dynamic and can be adjusted when more information is understood. They allow problem solvers to find solutions. We consider number processing to be part of building the mental representation, rather than being run only once the mental representation already exists.

Naturally, properties of the numbers used in word problems play an important role in word problem solving. Numerical properties such as parity and magnitude or the type of operations and numbers have rarely been thoroughly investigated independently from one another in word problems, despite their important role in current numerical cognition research (see Daroczy et al., 2015, for a review). Also, perceiving and semantically understanding multi-digit numbers is more challenging than single-digit numbers (Nuerk et al., 2011, 2015), and arithmetic complexity typically increases with the number of digits in standard arithmetic tasks. This phenomenon appears to hold true for word problems as well. For instance, Thevenot and Oakhill (2005) compared the influence of 3- and 2-digit numbers on word problem solution strategies and demonstrated that calculating more complex 3-digit numbers facilitated alternative strategies among the participants. However, the authors suggested that this facilitation was due to higher workload and working memory demands rather than due to the timing of number processing. Similar results were observed by Brissiaud and Sander (2010), who manipulated the size and order of the numbers and operations and found that number magnitude and order determined which strategy was most likely to be used.

However, not only the number of digits is relevant in a word problem. For example, children perform better if the numbers involve multiples of 25 (Koedinger & Nathan, 2004). Even when the number of digits is controlled, the complexity of place-value processing can influence word problem performance. Moreover, the carry and borrow effects play a role in word problems (e.g., Dresen et al., 2020). Carrying (in addition) occurs when the sum of digits in a column exceeds the base value (i.e., 10 in base-10 system), transferring the excess to the adjacent higher place value. Borrowing (in subtraction) occurs when a digit in the minuend is less than the corresponding digit in the subtrahend, necessitating the transfer of a unit from the next higher place value to proceed with the subtraction. RT have been found to be longer, and ER higher, in carry or borrow compared to non-carry or non-borrow addition and subtraction arithmetic problems (carry effect: Ashcraft, 1995; Moeller et al., 2011b; borrow effect: Artemenko et al., 2018). Similar carry and borrow effects have also been observed in word problems (e.g., Daroczy et al., 2020).

Apart from the number of digits and the necessity of carrying and borrowing, the problem size can also influence performance in word problems (De Corte, 1988) The larger the problem size, the slower the responses and the more errors are made. Furthermore, the operation to be carried out plays an important role in word problem solving, such that subtraction problems are more difficult than addition problems (e.g., Daroczy et al., 2020). Importantly, this is connected to the semantics of the word problems, so operations cannot be seen as purely numerical factors.

For the present work, number processing and text processing are used as rather broad terms. Number processing involves basic, automatic steps, such as recognizing numerical information and understanding the numbers’ magnitudes. Additionally, number processing includes integrating numbers into the mental representation, and potentially also selecting operations and performing calculations. Note that in the literature, the term number processing also sometimes refers to the mere understanding of the semantic meaning of numbers in terms of their magnitude, which has been shown to take place automatically even when not intended or required by the task (Tzelgov et al., 1992). In the current study, however, we expand this restrictive definition of number processing.

Taken together, numerical complexity in arithmetic word problems (e.g., number of digits in used numbers, necessity of a carry or borrow operation, problem size) plays an important role for performance. Although some studies acknowledge numerical effects in word problems (Daroczy et al., 2015, for an overview), there is uncertainty about whether number processing already happens in parallel to text processing, or whether number processing takes place only afterwards.

### Models explaining arithmetic word problem processing

Several types of models have been proposed to explain cognitive processing in arithmetic word problems. While some models propose sequential processing with different phases following one another, others propose interactive processing with different kinds of information being processed in parallel.

The first models explaining arithmetic word problem solving came up in 1984 and 1985 and were clearly sequential models. Mayer et al. (1984) described different phases that play a role in solving word problems. They stated that after the initial reading, the text is mentally represented, and this mental representation is then used to initiate the specific problem-solving process. One year later, Kintsch and Greeno (1985) published the Propositional Theory, which suggests that the mental representation is created based on a so-called propositional text base. According to this theory, which falls into the category of *schema models*, processing steps in problem solving are sequential. Schemas are stored in long-term memory, activated after the text processing, and then filled out with numbers (Kintsch & Greeno, 1985). In some types of word problems, using schemas as a strategy may be effective (Marshall, 1995), while in other cases, it may not work, and the situational context of a word problem might hinder the application of schemas (e.g., Stern & Lehrndorfer, 1992). Therefore, schema models has been challenged across many studies by *situation models* (e.g., Thevenot et al., 2007). Situation models suggest that an episodic situation model is created from the word problem, which is then translated into a mental representation. Currently, there is a consensus that a mental representation of the situation is indeed required (e.g., Pongsakdi et al., 2020). Ultimately, all these models can be seen as sequential models.

In a different theory, Bergqvist and Österholm (2010) proposed that reading and solving task demands are tightly integrated. The authors argue for a magnitude-based, more dynamic mental representation. Dynamic mental representations of word problems would be in line with conclusions drawn from studies on text processing (in terms of reading comprehension in non-mathematical stimuli), where mental representations have been claimed to be updated regularly and where inferences have been shown to be made on-line while reading (Gernsbacher et al., 1992, 1998). In contrast to sequential models by Mayer and colleagues (1984) and by Kintsch and Greeno (1985), the model by Bergqvist and Österholm is clearly interactive, with different parts of cognitive processing happening simultaneously.

A more recent model, which can be understood as an extension of situation models, is Gros et al.’s (2020) semantic congruence model (SECO). It identifies three factors crucial for solving word problems: mathematical semantics (understanding math concepts), algorithms (strategic problem-solving), and world semantics (real-world knowledge). The SECO model also integrates knowledge about the semantic relations between the problem elements and the mental representations of the problems (e.g., Bassok et al., 1998). The nature of mental representations and the influence of semantic congruence have recently been demonstrated by a thoughtfully designed study, where participants were not only asked to solve, but also to recall the word problems in an unexpected memory task (Gros et al., 2024). Importantly, in the SECO model, mathematical knowledge seems connected to language rather than to number processing such as addition and subtraction. The SECO model also extends previous models with a feedback loop. Some models, especially from educational research (e.g., Blum & Leiss, 2005), also use a feedback loop. However, in this case, the existing mental representation is renewed after an incorrect strategy, and these models do not sufficiently explain what happens when only the numerical difficulty is altered, without any connection to the semantics of the text. The SECO model does not make a clear statement about whether cognitive processing of the three postulated factors (i.e., mathematical semantics, algorithms, and world semantics) takes place sequentially or in parallel. However, the SECO model includes a possible recoding loop which relies on mathematical semantics. This recoding loop can be activated in the case of semantically incongruent information to correct the original mental representation, making the model non-sequential.

An analogy to the relation between text and number processing in mathematical word problems is the relation between syntax and semantics in language. On the one hand, language establishes the structure of the problem, and on the other hand, it allows inferences about the numerical problem (for an overview see Singer, 1994). Such inferences are not necessarily logical, but can also be pragmatic (Harris & Monaco, 1978; Singer, 1988). In fact, in non-standard word problems, most inferences are not strictly logical, but pragmatic in the sense that they rely on people’s common knowledge about the world. For instance, a popular non-standard word problem is: “Ten birds are sitting on a tree. The hunter shoots down two birds. How many birds are still on the tree?”. The logical inference (without using world knowledge) would be that 8 birds are left, because only 2 of the 10 birds are shot. The pragmatic inference, which many children and adults do not use in such word problems, is that when some of the birds are shot, all other birds fly away. In the 1990s and afterward, significant research examined the relationship of syntax and semantics in language (van Valin, 2005). Both play a critical role in word problems. For instance, Daroczy et al. (2020) showed that both syntactic (e.g., nominalization) and semantic features (e.g., lexical consistency of keywords) play a role in understanding and solving word problems. Hence, in a similar way the interaction of syntax and semantics was studied before, we now investigated the interplay between text processing and number processing in word problems. We believe that text processing and number processing are as deeply intertwined as syntax and semantics in text and discourse processing.

To sum up, some models about cognitive processing in arithmetic word problems are sequential and assume that number processing happens only after text processing. According to other models, number processing may occur in parallel with text processing. Moreover, some models are agnostic about when number processing takes place when solving arithmetic word problems. The aim of the current study was to determine whether number processing occurs at an early stage, thereby contrasting sequential and interactive models of word problem-solving.

### Number processing in models explaining arithmetic word-problem processing

After this overview of the models explaining arithmetic word-problem processing, we would like to revisit them in terms of how they address number processing within arithmetic word-problems. Different models interpret and utilize numbers in distinct ways, giving them different roles and importance. However, most models assign limited importance to numbers. Instead, most studies identify semantic and linguistic factors, rather than numerical ones, as the reasons why people fail to construct a correct mental representation of the problem. The review by Jaffe and Bolger (2023) argues that cognitive load increases when numbers are presented as text rather than in Arabic format; the reason for this is that number processing cannot circumvent a phonological step and be directly mapped onto numerical values (Damian, 2004). This suggests that number processing might interact with other problem-solving steps, but the review does not address the possibility of a magnitude-based mental representation.

In contrast, the schema model (e.g., Kintsch & Greeno, 1985) is rather sequential. Here, the emphasis lies on first activating schemas in long-term memory and afterwards instantiating them with specific numerical values. Hence, numbers only play a role after the conceptualization, indicating a more significant role of linguistic features and reading. In line with this, Fuchs et al. (2018) describe text processing and number processing in word problems as two successive phases. Specifically, in the first phase, the text’s essential ideas are captured to build up an adequate mental representation of the problem. This includes a semantic understanding of the words and potentially also of the numbers (i.e., the extraction of their magnitude, which is highly automatic; e.g., see Tzelgov et al., 1992). In the second phase, the solution-relevant mathematical operations are deduced from the representation and calculations are performed by executing the chosen operations. According to this, numbers seem to play a role in the later stages, aligning with the last step of problem-solving.

The SECO model (Gros et al., 2020) partly incorporates the role of numbers in one of its essential components, namely mathematical semantics. Mathematical semantics is defined as the solver’s mathematical knowledge needed for the given problem statement, which enables the identification of solving algorithms. The SECO model therefore indirectly addresses the challenges associated with numbers in arithmetic word problems, but it does not explain why problem-solving performance changes when the numbers are changed (e.g., in the case of carry/borrow), particularly when the numbers are altered in a way that does not affect the underlying mathematical semantics.

Across these models, a common step is the translation of the interpreted structure into a mathematical operation, which is assumed to occur at a later stage. Interestingly, the choice of words in the text is claimed to have a significant influence on this translation. However, the models do not adequately address the impact of choice of numbers.

In contrast, Bergqvist and Österholm (2010) argue that number processing is highly integrated and cannot be separated from other problem-solving processes such as text processing. According to their model, heuristic approaches allow number processing to start during the initial reading and argue for a magnitude-based mental representation, however their assumption was not empirically proven. There are indeed very few empirical studies investigating the existence of magnitude-based mental representations. For instance, Orrantia and Múñez’ (2013) study provided evidence that an analog magnitude-based mental representation is consistently activated during word-problem solving. In their study, participants solved word problems and then completed a figure discrimination task. Participants demonstrated slower discrimination when the magnitude relationship between figures did not align with the implied quantities' relationship in the sentence they read. However, a potential criticism emerges concerning whether participants formed a genuine representation of magnitude: The figures used in the discrimination task only reflected the relationship between variables in the relational sentence (e.g., John > Peter), failing to capture relative magnitudes based on the numbers presented in the word problem. It is crucial to note that these findings specifically pertain to compare problems and do not extend to change problems. Compare word problems involve evaluating the differences or similarities between two or more items, quantities, or scenarios to understand their relationship better. Change word problems deal with understanding how a quantity increases or decreases over time or through a series of events, focusing on the transformation from one state to another. Additionally, the measurements were taken post problem-solving rather than during the problem-solving process itself, which is an issue we aim to address.

It remains unclear whether aspects determining the numerical complexity of word problems that we discussed above speak for sequential or interactive models of word problems. The question arises whether more demanding and error-prone calculations only manifest their effect on performance in later processing stages (i.e., in line with models favoring sequential processing phases), or whether they already influence performance at an earlier stage (i.e., in line with models favoring interactive processing phases). As stated above, one methodological problem is that many studies rely only on conventional outcome variables such as RT and ER, which allow to understand the final phase of the word-problem solving process, but do not allow us to dive into the process itself. In contrast, eye-tracking can help to reveal mental representations and cognitive processes during the solving process which cannot be detected by analyzing the final phase of behavior and can also not always be reported consciously (De Corte & Verschaffel, 1986).

We hypothesize that number processing begins early and simultaneously with text processing, where the text is read and mentally conceptualized. To test this hypothesis, we used a straightforward concept: In the present study, we tested whether individuals looked more often or for longer durations at numbers in word problems involving more complex calculations compared to word problems with simpler calculations, even when no calculation was necessary. For this purpose, we designed solvable and non-solvable word problems. In the latter ones, participants had to determine that the word problem is non-solvable. Non-solvable word problems fall into the category of non-routine or non-standard word problems. Such problems cannot be solved using known methods or formulas (Artut, 2016), their solutions are not immediately apparent (Celebioglu et al., 2010; Murdiyani, 2018), and success often relies more on logical thinking than on traditional mathematical knowledge and skills acquired in school (e.g., Abdullah et al., 2014). To realize that the word problem lacks a solution, participants do not need to consider the numbers; they aredispensable, at least in the present study. Please note that certain types of non-solvable word problems may require individuals to perform calculations to realize that they are non-solvable. In such cases, number processing is necessary (e.g., “John has 13 apples. Mary has 36 fewer. How many apples does Mary have?”). However, in our study, we focus exclusively on non-solvable word problems where number processing is not needed to determine that the problem is non-solvable. However, participants might engage in the direct translation strategy described by Hegarty et al. (1992, 1995), using the numbers and keywords to perform a calculation. Outcomes from this approach will be excluded from the analysis because the mental representation of a non-solvable word problem must be incorrect if a calculation is carried out. It is possible that individuals begin with the direct translation strategy and later recognize that the problem is non-solvable. They might then alter their mental representation of the word problem or give up. However, even in giving up, there’s a recognition of a mismatch with their initialproblem representation, indicating at least partial integration of text processing (including the creation of a mental representation) and number processing (including potential calculation). Note that even if people realize the problems are non-solvable and create the correct problem representation, we will still not know when and how they use the numbers. Therefore, we manipulated the complexity of the calculation, specifically the necessity of carrying or borrowing, by including problems that contained either the simpler or the more complex hypothetical calculation. Theoretically, if a correct mental representation of the situation is created, and people realize in time that number processing including calculating is unnecessary, they will not use their cognitive resources for calculations. This would only happen if number processing was integrated from the very beginning.

### Eye-tracking in word problem research

To adequately investigate the cognitive processes during solving arithmetic word problems, the method of eye-tracking has proven to be useful for word problems in numerous studies (e.g., De Corte et al., 1990; Hegarty et al., 1992; Knoblich et al., 2001). Eye-tracking data have been demonstrated to be reliable and valid (e.g., Lai et al., 2013; Susac et al., 2014). Eye-tracking methodology appears to be particularly valuable for studying processes, as demonstrated by Strohmaier et al. (2020). It can help reveal mental representations and assess mathematical cognitive processes that cannot be consciously reported (De Corte & Verschaffel, 1986). The central assumption of eye-movement analysis is the eye-mind assumption, which posits that participants look at the element that is currently being processed (Just & Carpenter, 1980). Consequently, the fixation on words should indicate current text processing, whereas the fixation on numbers should indicate current number processing. Please note that the eye-mind assumption has been challenged several times, and it has been shown that people do not necessarily have to look at a specific area to process information. Nevertheless, Moeller et al. (2011b) found significantly longer attention on carry addition problems compared to non-carry addition problems, suggesting that increased attention on numbers is a valid reflection of deeper processing in a specific area.

However, the question is how these metrics are related to different parts and stages of word problem solving. An important distinction to be made is between static and dynamic eye-tracking variables. People can fixate, jump to, and revisit specific areas in word problems. Lai et al. (2013) and Strohmaier et al. (2019) define fixations as relatively stable states of eye movement and saccades as rapid, straight movements to the next fixated point. Regressions can be understood as all fixations made after the respective area has been left for the first time; they are relatively stable states of eye movement after the first visit. Fixation duration (FD), fixation count (FC), regression duration (RD), and regression count (RC) are the most common measures in word problem solving research (Strohmaier et al., 2019). These four eye-tracking variables are static variables because they describe stable states of the eye, rather than dynamic eye tracking variables, which describe movements from one area to the next. FC and FD reflect the amount of attention invested and are related to processing difficulty (van der Schoot et al., 2009). To obtain the RD or RC, the first-pass FC or FD within the areas of interest (AOI) is subtracted from the total FD or FC (Lai et al., 2013). Regressions to numbers might also reflect number processing, in terms of the execution of the solution-relevant mathematical operations. This is also supported by Dresen et al. (2020) who found significantly longer durations of first fixations and regressions in carry addition problems compared to non-carry addition problems. Therefore, we expect to see similar differences in these measures between carry and non-carry contexts in solvable and even non-solvable word problems.

In previous word problem studies, mostly static eye-tracking measures were applied. Nevertheless, eye movements have a dynamic character, and the eyes move from one fixation to another with rapid movements (i.e., saccades). These movements can be analyzed via scan paths (e.g., Jian & Wu, 2015) or sequence charts (e.g., Strohmaier et al., 2018), for example. Scan paths are sequences of readers’ fixations and saccades. Most of these measures are exploratory in mathematical cognition, and it is challenging to link eye movements to cognitive processes. To clarify the relationship between text and number processing, it is important to examine dynamic shifts occurring between the text and the numbers. Eye movements (i.e., transitions) between text parts, between numbers and text, and between numbers can be seen as indicators for reading comprehension, calculation, and overlapping processes. Movements between text and numbers could suggest that these elements are combined and considered together (Johnson & Mayer, 2012). Regressions to previously read text often signals reading comprehension (e.g., Rayner, 1998; Verschaffel et al., 1992), whereas returning to numbers suggests active engagement in calculation (Hegarty et al., 1995). In eye-tracking research, transitions have been analyzed in various studies, though many focus on graphic reading (e.g., Malone et al., 2020) or fields such as chemistry (Rodemer et al., 2020). However, they have not yet been extensively studied in the context of word problems and mathematical cognition.

In summary, eye-tracking is a valid method for measuring cognitive processes in arithmetic word problems. If the number processing plays a role, attention (fixations, regressions, and transitions) should be drawn to areas of the problems containing numbers.

### Purpose of the present study

The goal of the present study is to investigate whether number processing (i.e., recognizing numbers, semantically understanding their magnitude, and potentially performing calculations) in arithmetic word problems occurs simultaneously with text processing or only afterwards. The research specifically examines eye movements, analyzing if people look more often or for longer periods at numbers in more complex problems than in simpler ones, even when a calculation is not needed and participants are aware of this (i.e., build the correct mental representation). If number processing occurs simultaneously with other problem-solving processes such as text processing, there will be an increase in attention (i.e., fixations and regressions on the numbers, and more transitions between numbers or between numbers and text parts) in the non-solvable word problems involving carry or borrow addition or subtraction operations as compared to the non-solvable word problems involving non-carry and non-borrow operations, even when participants categorize them correctly as non-solvable.

Furthermore, we anticipate that the same patterns will emerge in solvable word problems, which require calculations. Like in our pilot study with children (Roth, 2021), we expect lower ER in solvable word problems compared to non-solvable ones, replicating the findings of Jiménez and Verschaffel (2014) and Verschaffel et al. (1994). Additionally, we anticipate longer RT in solvable problems due to the time needed for calculations. Within the category of solvable word problems, we hypothesize that the carry or borrow condition will result in longer RT and higher ER compared to the non-carry or non-borrow condition, as indicated by many studies (e.g., Artemenko et al., 2018; Ashcraft & Stazyk, 1981; Daroczy et al., 2020; Moeller et al., 2011b; Radler et al., 2014). As previous studies have shown higher RT in subtraction compared to addition problems (e.g., Daroczy et al., 2020; Radler et al., 2014), suggesting that subtraction is more challenging than addition, we expect to find higher RT and ER in solvable subtraction compared to addition problems in our study as well. Similarly, regarding the measured eye movements, we hypothesize more and longer fixations and regressions on the numbers (AOI) as well as more transitions between numbers or between numbers and text parts in solvable subtraction than addition problems.

Understanding the sequence of cognitive processes in word problem solving, specifically the role of number processing relative to the formation of a mental representation, is crucial for effective education and cognitive research. If number processing occurs simultaneously within the formation of a mentalrepresentation, educators should not only focus on enhancing both text comprehension and numerical skills, but also be aware that teaching a strictly sequential process does not correspond to how healthy adults with an average performance level solve word problems. Nevertheless, children may rely on a more sequential strategy than adults. As children develop proficiency, they may gradually shift towards more simultaneous text and number processing, becoming more similar to the typical strategy used by adults. An analogous case can be seen in strategies for calculation tasks with distractors: adults show flexibility in choosing correct answers or distractors, whereas children consistently select distractors (Moeller et al., 2011a, 2011b). While this pertains to calculation tasks, similar differences may be conceivable in word problems. Understanding the sequence of cognitive processes in arithmetic word-problem solving may eventually help in tailoring beneficial and avoiding inefficient teaching strategies, enhancing students’ performance.

## Method

This study was approved by the Ethics Committee of the Department of Psychology.

### Participants

The sample consisted of 63 adults (*M*_*age*_ = 23.14, *SD*_*age*_ = 5.87, *N*_*female*_ = 42, *N*_*male*_ = 21), who were recruited through a mailing list and a recruitment system at the University of Tübingen as well as through private contacts of the investigators. Inclusion criteria required participants to be at least 18 years old, have German as their native language, and have no neurological disorders. 58 participants were right-handed, and five participants were left-handed. None reported dyslexia or dyscalculia, and 14 participants needed glasses or contact lenses during the experiment. University students in Psychology or Cognitive Science were granted 1.5 h of course credit for their participation.

### Stimuli and design

#### Arithmetic word problems

The study followed a 2 (solvability: solvable vs. non-solvable) × 2 (difficulty: simple vs. complex) within-subject design. In non-solvable word problems, questions either referred to an aspect that was previously not mentioned in the word problem, or to a previously mentioned aspect with the information about that aspect being insufficient to answer the question (as described by Jiménez & Verschaffel, 2014). In solvable word problems, in contrast, questions referred to a previously mentioned aspect and sufficient information was given for questions to be answered. Correct solutions of solvable word problems were two-digit numbers. Difficulty was operationalized by the necessity of a carry or borrow operation. Simple word problems were non-carry addition and non-borrow subtraction word problems (e.g., non-solvable addition of 72 + 26 and solvable subtraction of 59 − 25 in Table 1), while complex word problems were carry addition and borrow subtraction word problems (e.g., solvable addition of 67 + 26 or non-solvable subtraction of 56 − 27).

**Table 1:** Four word problems as an example for parallel word problems constructed from the same story

Difficulty	Solvability	
	Solvable	Non-solvable
Simple	Max und Leon spielen mit Murmeln. *(Max and Leon are playing with marbles.)* Max besitzt 59 Murmeln. *(Max owns 59 marbles.)* Er hat 25 mehr als Leon. *(He has 25 more than Leon.)* Wie viele Murmeln hat Leon? *(How many marbles does Leon have?)*	Max und Leon spielen mit Murmeln *(Max and Leon are playing with marbles.)* Max besitzt 72 Murmeln *(Max owns 72 marbles.)* Er hat 26 weniger als Leon *(He has 26 less than Leon.)* Wie viele Murmeln gibt er Leon? *(How many marbles does he give to Leon?)*
Complex	Max und Leon spielen mit Murmeln. *(Max and Leon are playing with marbles.)* Max besitzt 67 Murmeln. *(Max owns 67 marbles.)* Er hat 26 weniger als Leon. *(He has 26 less than Leon.)* Wie viele Murmeln hat Leon? *(How many marbles does Leon have?)*	Max und Leon spielen mit Murmeln. *(Max and Leon are playing with marbles.)* Max besitzt 56 Murmeln. *(Max owns 56 marbles.)* Er hat 27 mehr als Leon. *(He has 27 more than Leon.)* Wie viele Murmeln gibt er Leon? *(How many marbles does he give to Leon?)*

All four word problems are lexically inconsistent. The presented examples illustrate one version of the solvable simple word problem, which requires subtraction, and the solvable complex word problem, which requires addition. Similarly, one version of the non-solvable complex word problem seems to require subtraction, and the non-solvable simple word problem seems to require addition. However, there are cases where not only subtraction but also addition may be involved, and vice versa

Arithmetic operation was counterbalanced, so that half of the word problems in each condition were addition and subtraction word problems to ensure that participants kept reading the text, as recommended by Daroczy et al. (2020). In contrast, we decided not to fully counterbalance difficulty to have only four (each combination of solvability and operation) instead of eight (each combination of solvability, operation, and difficulty) word problems based on the same story. Only single-step word problems were used in the present study. Each word problem consisted of four sentences: a first introductory sentence, a second sentence containing the larger number, a third sentence containing the smaller number, and a fourth sentence being the question to be answered. Note that alternatively, the question could have been placed at the beginning of the word problem, which can help solvers to construct a mental representation according to Thevenot et al. (2007). This particularly holds for compare problems, where relationships are clear from the question (e.g., “How many more marbles does John have than Mary?”). However, this might not apply to change problems, where understanding the full context is essential, and the question may not contain every information. Placing the question at the beginning may also reduce cognitive load by directing attention to the problem’s goal and thus allowing learners to focus on relevant numbers right away. On the other hand, presenting the question at the end requires solvers to engage with the entire problem content first, encouraging a more comprehensive understanding of both numerical and contextual details like in real-life situations, which the current study aimed at. The questions were about different aspects, such as persons, objects, actions, times, locations, animals, or units. Each word problem existed in four parallel versions according to experimental conditions, as illustrated in Table 1 with an example: one solvable addition, solvable subtraction, non-solvable addition, and non-solvable subtraction word problem each.

In the construction of the word problems, potential linguistic confounds were carefully controlled. First, the word problems did not include potentially incomprehensible expressions or linguistic ambiguities. Second, all included first names were part of the ten most frequently given male and female names in Germany in 2010 (Gesellschaft für deutsche Sprache 2011), which was the close to the birth year of the participants in the current study. Third, the number of words and letters per word problem as well as the total frequency of use of all employed words (determined within the database Corpora by Schäfer, 2015; Schäfer & Bildhauer, 2012), which can influence word problem difficulty (Haag et al., 2013), did not significantly differ between solvable and non-solvable, between simple and complex, and between addition and subtraction word problems (tested with two-sample t-tests). Fourth, since lexically inconsistent word problems (when the keyword matches the required operation, e.g., when “more” appears in an addition word problem or when “less” appears in a subtraction word problem) are more difficult than lexically consistent word problems (e.g., when “loose” appears in an addition word problem or when “win” appears in a subtraction word problem; Daroczy et al., 2015, 2020), the same number of inconsistent and consistent word problems was included per condition.

Moreover, mathematical features and characteristics of the used numbers that typically influence the difficulty of multi-digit addition and subtraction (for an overview, see Nuerk et al., 2015) were carefully controlled. First, we accounted for the problem size effect (Brysbaert, 1995; Moeller et al., 2011b), which describes increasing difficulty with increasing number magnitude, by keeping the average of the first and second number constant (*M*_*first number*_ = 42.5, *M*_*second number*_ = 20.0). Thus, the average solution in addition (*M*_*problem size*_ = *M*_*sum*_ = 62.5) and subtraction word problems (*M*_*difference*_ = 22.5) was equal between conditions. Second, the odd effect was taken into consideration, which describes faster responses to even than to odd numbers and seems to be even more pronounced in two- than in single-digit numbers (Hines, 1990). To control for the odd effect, the parity status of numbers was counterbalanced between conditions: Each condition (e.g., solvable simple word problems) contained one word problem with two even numbers, one word problem with two odd numbers, one word problem with the first number being even and the second number being odd, and one word problem with the first number being odd and the second number being even. Third, we controlled for the decade crossing effect, according to which number processing is facilitated when the decade of two multi-digit numbers is identical (Nuerk et al., 2015), by ensuring that the two numbers used within one word problem never had the same unit or decade. Fourth, the decade number effect, referring to multiples of ten (e.g., 40) being processed faster than other numbers of a similar magnitude (Nuerk et al., 2015), was controlled for by not using decade numbers within the word problems or as correct solutions. Finally, no numbers with an identical unit and tens digit (e.g., 22) were included within the word problems or as correct solutions, and one number never consisted of the reversed digits of the other (e.g., 51 and 15) within a word problem. The entire stimulus set can be found in Supplementary Material A.

#### Filler tasks

Besides the 32 carefully manipulated word problems described above, the stimulus set comprised 16 filler tasks, which were not relevant for the research question (Supplementary Material B). Filler tasks were included as distractors to prevent participants from getting used to the structure of the relevant word problems and not included in the data analysis. Therefore, as opposed to the relevant word problems, some filler tasks required more than one step, some included more than two numbers, some included numbers that were no two-digit numbers, and some of them required other arithmetic operations than addition or subtraction. Just as the relevant word problems, filler tasks consisted of four sentences, however, all of them were solvable. There were two parallel versions of each word problem with the same story, namely one routine word problem and one non-routine word problem. The latter followed an uncommon structure or included an unexpected question. For example, the non-routine filler task in Table 2 does not require calculating at all because the answer is included in the text.

**Table 2:** Two word problems as examples for parallel filler tasks constructed from the same stories

Type of filler task	
Routine	Non-routine
In einem Restaurant stehen Kerzen auf den Tischen. *(In a restaurant, there are candles on the tables.)* Es brennen bereits 42 Kerzen. *(There are 42 candles already burning.)* 26 Kerzen sind noch nicht angezündet. *(26 candles are not yet lit.)* Wie viele Kerzen stehen insgesamt auf den Tischen? *(How many candles in total are on the tables?)*	In einem Restaurant stehen 52 Kerzen auf den Tischen. *(In a restaurant, there are 52 candles on the tables.)* Es brennen bereits 31 Kerzen. *(There are 31 candles already burning.)* 21 Kerzen sind noch nicht angezündet. *(21 candles are not yet lit.)* Wie viele Kerzen stehen insgesamt auf den Tischen? *(How many candles in total are on the tables?)*

Both filler tasks are solvable, but the first is a routine and the second is a non-routine word problem. The routine word problem requires addition, whereas no arithmetic operation is required to correctly answer the non-routine word problem

#### Cognitive covariates

After the word problems, we administered a battery of neuropsychological tests to the participants with the primary aim of confirming that our sample accurately reflects a normal population. The inclusion of these tests allowed for a comprehensive assessment of cognitive functioning, ensuring that our findings are grounded in a representative cross-section of individuals without discernible neurological or cognitive impairments. First, figural, verbal, and numerical creativity were assessed. Next, basic mathematical competencies were tested by two speed calculation tests. Last, the capacity of participants’ verbal and spatial working memory was tested. The assessment of cognitive covariates is described in more detail in the Supplementary Material C.

### Procedure

The study took place in a laboratory at the University of Tübingen, Germany. To avoid daylight fluctuations altered participants’ pupil size during the eye-tracking part, windows were entirely covered. Depending on what worked better in the eye-tracker calibration, the experimenter decided for each participant individually in the very beginning whether to turn on or off the light in the laboratory, and these light conditions were kept stable during the eye-tracking procedure.

In a first step, participants were informed and consented to participate. For this, they were told that their eye movements would be recorded by an eye-tracker, which is an absolutely pain and risk-free method, and that their vocal answers to word problems would be recorded. After giving written consent and filling in a demographic questionnaire (age, gender, handedness, native language, diagnoses of dyslexia or dyscalculia, corrected vision, dominant eye, years of studies, study field or occupation). Participants were asked to sit on a chair in front of a desk in a comfortable position and to move as little as possible while solving the word problems. The microphone and the monitor with the eye-tracker were placed on the desk approximately 20 cm away from their mouth and 50 cm away from their eyes, respectively. To ensure that the participants could continually look at the screen, participants were asked to respond vocally to the word problems. RT in the experiment were measured from the word problem’s appearance on the screen to the vocal response. To ensure the voice key would be triggered and RT would be recorded similarly for all word problems, participants were instructed to start every response with the German word “gleich” (English: “equals”) and to speak loudly, while not making any other noise. To familiarize participants with the setup, to get them used to starting each answer with “gleich”, and to adjust the sensibility of the microphone, participants were asked to answer 11 personal questions (e.g., “Do you have a pet?”), reading out loud 11 numbers, and responding to five solvable practice word problems.

After these practice trials, a target sticker was placed in the center of the participant’s forehead as a reference point for the eye-tracker. We employed monocular eye-tracking for the dominant eye, identified either through participant´s self-report or by assessing eye dominance via a method involving looking through a hand-formed hole and comparing the dominance of the images produced by the left and right eyes. Next, instructions appeared on the screen, in which participants were asked to mentally calculate the solution for the displayed arithmetic word problems as fast and as accurately as possible and say their response out loud while starting with the word “gleich” (English: “equals”). In case of non-solvable word problems, participants were instructed to say “gleich Aufgabe nicht lösbar” (English: “equals problem not solvable”). Participants were asked to look at the fixation point appearing centered in the upper part of the screen after each word problem. Participants were informed that if they would not answer, the next word problem would appear after a while, and that the total word problem test would take around 25 min with a short break halfway through. Next, for the eye-tracker calibration, participants fixated nine points appearing one after the other on the screen. The calibration was validated by repeating the procedure until the deviation between the fixations in a first and second round did not deviate by more than 1.5 degrees for each point, or less than 1.0 degrees averaged over all nine points. The calibration was validated again before each word problem by the participants looking at the fixation point. If the fixation was not recognized correctly, the eye-tracker was recalibrated as in the beginning.

Word problems were presented in white Times New Roman font in 24 pt size on a black background. All four sentences appeared simultaneously centered on the screen with one sentence per line. As soon as the verbal response triggered the voice key or after a maximum of 90 s, the word problem was replaced by a blank black screen. After three seconds, a black and white noise screen appeared for 60 ms, followed by the fixation point. Reponses were both noted down by the experimenter and recorded. The 48 stimuli (i.e., 32 relevant word problems and 16 filler tasks) were presented in two blocks with a break halfway through. The number of solvable vs. non-solvable word problems, of simple vs. complex word problems, of addition vs. subtraction word problems, of word problems with the same story, and of routine vs. non-routine filler tasks were equal in each block. Block order was counterbalanced between participants, and stimulus order was fully randomized within blocks.

In the second part of the study, participants completed questionnaires and tests in a paper–pencil format to assess cognitive covariates that are typically related to the performance in arithmetic word problems. By assessing cognitive characteristics of the sample, potential anomalies are detectable, and the study is more replicable. Moreover, it provided the opportunity to validate correlations between the measured eye-tracking variables and these cognitive covariates. First, figural, verbal, and numerical creativity were assessed by the LO subtest of the BIS-HB (Jäger et al., 2006), the ASK (Schuler and Hell, 2004), and the DR1 subtest of the BIS-HB, respectively. Afterwards, participants’ basic mathematical competencies were assessed by two speed calculation tests (Huber et al., 2013). Finally, verbal and spatial working memory capacity was tested with the letter span task and the Corsi Block-Tapping Test (Corsi, 1972), respectively. In total, participation in the study took between 75 and 90 min. Results for all cognitive covariates assessed in the second part of the study are summarized in the Supplementary Material C.

### Software and material

The word problem part of the study was programmed with the *Experiment Builder *(SR Research Ltd., version 2.1.4) running on a first computer. Stimuli were presented on a 19-inch monitor with a resolution of 1025 × 768 pixels. Eye movements were recorded via *EyeLink 1000* (SR Research Ltd., Mississauga, Canada), an infrared video-based tracking system, using a 500 Hz sampling rate and a 16 mm lens. The eye-tracker was connected to a second computer displaying the eye-tracking software and the participant’s eye movements on a second monitor for the experimenter at any time. Eye-tracking data was stored and later preprocessed for the data analysis in the *Data Viewer* (SR Research Ltd., version 3.2.1. Vocal responses as well as RT from stimulus presentation to the triggered voice key were recorded with the help of the *EMU 0202 USB Audio Interface* (Creative Technology Ltd., Singapore). Moreover, a pen and copies of the informed consent as well as the questionnaires and tests for cognitive covariates were needed.

### Dependent variables

Number and text processing were operationalized by the attention to the numbers and to the text in the word problems, as reflected both by static and dynamic eye-tracking measures. For this, we created rectangular AOI around the two numbers within the 32 word problems (height of 136 pixels, width of 40 pixels wide) in the eye-tracking software. The two numbers were always located in the second (y-coordinates of 246 and 382 pixels) and third line (y-coordinates of 386 and 522 pixels), but the location within these lines varied (x-coordinates differed depending on the word problem). On top of static and dynamic eye-tracking measures, we also recorded RT and ER.

We chose not to treat the reading of the question (i.e., first or last fixation on the question) as a the starting time point of measurement because reading might be non-linear (i.e., eye movements moving back and forth) and because of our hypothesis that word-problem solving does not necessarily follow a strictly sequential process. That is, by using an exclusion criterion for all stimuli where participants have not read the entire question, we might have risked considerable data loss. Furthermore, judgments about solvability and overall comprehension rely on integrating information from all parts of the problem.

Moreover, based on our study manipulation, we cannot determine when people recognize that a problem is non-solvable, nor can we separate how many eye movements happen before or after this time point. This would require a different type of experimental manipulation and follow-up research.

#### Static eye-tracking measures

The four measured static eye-tracking variables were FD, RD, FC and RC. FD was defined as the total duration of all stable states of eye movement within the two AOI. RD was defined as the total duration of such stable states from the second fixation on, that is, after AOI were left for the first time in the respective word problem. FD and RD were measured in milliseconds (ms). FC and RC were defined as the total number of stable states of eye movement within the two AOI and after AOI were left for the first time, respectively.

#### Dynamic eye-tracking measures

The measured dynamic eye-tracking variables were three different types of transitions. Transitions are saccades, or rapid eye movements, that occur either between or within an AOI. For this, we defined three areas: (i) The first number appearing in the second sentence of the word problem, and (ii) the second number presented in the third sentence of the word problems correspond to the AOI used in static eye-tracking measures (referenced above). Moreover, (iii) the text was defined as all remaining textual content of the word problems (x-coordinates of ABC and DEF pixels, y-coordinates of ABC and DEF pixels).

Table 3 categorizes the transition types into number-to-number (NN), text-to-text (TT), and text-to-number, or number-to-text (TN), along with their descriptions and possible interpretations in the context of number and text processing within word problems. Note that in the present study, we did not differentiate between text-to-number and number-to-text transitions, because both transition types occur in case the first initial reading phase is sequential (i.e., as the two numbers appear in the text, the pattern would be: text-to-number, number-to-text, text-to-number, number-to-text). However, this is not analyzed in detail in the current study, because we were interested in the general increase of TN transitions, which should reflect parallel number and text processing. Fixations may also occur outside of the defined interest areas but still on the main screen. These cannot be directly associated with specific cognitive processes within word problem-solving, and therefore, we do not have a specific hypothesis for them.

**Table 3:** Overview of the three measured types of transitions representing the dynamic eye-tracking variables in the present study, and their respective interpretations

Transition name	Eye movement	Interpretation
Number-to-number (NN)	Number 1 to Number 2 Number 2 to Number 1	Calculation
Text-to-text (TT)	Movements within the text, forward or backward	Reading comprehension
Text-to-number or number-to-text (TN)	Text to Number 1 Text to Number 2 Number 1 to text Number 2 to text	Interaction of calculation and reading comprehension

#### Performance measures

On top of eye-tracking measures, which were required to answer the main research question and thus our focus in the data analysis, we also recorded performance measures. On the one hand, RT were measured for each word problem separately as the time in seconds elapsing between the word problems’ appearance on the screen and the triggering of the voice key by the participants’ response. On the other hand, ER in terms of the proportion of incorrect responses for the 32 word problems were calculated for each participant separately. Responses to solvable word problems were correct if they were the exact solution answering the question, and responses to non-solvable word problems were correct if they stated that the word problems were non-solvable.

### Data preprocessing

After extracting the seven dependent eye-tracking variables (static: FD, RD, FC, and RC; dynamic: NN, TT, and TN) and the two performance measures (RT and ER) from the *EyeLink Data Viewer*, data preprocessing was performed in the statistical computing software R (R Core Team, 2021). Missing and incorrect responses led to exclusion of the respective trial for the analysis of all dependent measures except ER. Moreover, all parallel word problems constructed from the same story were excluded if less than 33% of the solvable or non-solvable versions were correctly answered, because this indicates that the word problem leads to misunderstandings or is too difficult.

### Data analysis

As for the data preprocessing, we used the statistical computing software R (R Core Team, 2021) for the data analysis, including the R packages *dplyr* (Wickham et al., 2023b), *ggplot2* (Wickham et al., 2023a), *ggpubr* (Kassambara, 2023), and *lmerTest* (Kuznetsova et al., 2017). The anonymized datasets and all R scripts for the data preprocessing and analysis can be found on the Open Science Framework (https://osf.io/nvdhq/).

#### Linear mixed models for static and dynamic eye-tracking and performance measures

To investigate potential interaction and main effects of difficulty and operation on the four static eye-tracking measures (FD, RD, FC, and RC), on the three dynamic eye-tracking measures (NN, TT, and TN), and on performance measures (RT, and ER), nine LMMs were fit for non-solvable word problems. For all measures except ER, only correctly answered word problems were considered. The nine equivalent LMMs for solvable word problems can be found in the Supplementary Materials D (static eye-tracking measures), E (dynamic eye-tracking measures), and F (performance measures). Moreover, for non-solvable word problems, the same analyses were run for FD and FC for the entire computer screen instead of only AOIs and results can be found in the Supplementary Material G.

Eye movements and performances can largely differ between participants, which is why we included random intercepts for the participants in each LMM. Eye movements and performances can also vary between word problems, but we did not expect this variance to be impactful because we carefully controlled linguistic and numerical features of the word problems we used, so that their difficulty should be approximately equal. Because participants might not carefully read the text in the word problems if there are too many repetitions, we decided not to fully counterbalance our design and have only four (combination of solvability and operation) instead of eight (combination of solvability, operation, and difficulty) parallel word problems based on the same story. Hence, difficulty was not counterbalanced within but between used stories (see Supplementary Materials A and I), so that the resulting 32 word problems were not independent from each other and a random intercept could not be included for them.

To sum up, for each of the nine dependent variables, the following model was fit with the function *lmer* from the *lmerTest* R package, while setting the argument REML = FALSE to run Maximum-likelihood estimation for the fixed effects:

$$Y_{ij} = \beta_{0} + S_{Pi} + \beta_{1} *difficulty + \beta_{2} *operation + \beta_{{3}} *difficulty \, * \, operation + \varepsilon_{ij} .$$

In this model equation, the criterion variable *Yij* can be replaced by each of the dependent variables (FD, RD, FC, RC, NN, TT, TN, RT, or ER). The model parameters for fixed effects were denoted with *β*, while random effects were denoted with *S* (as in Barr et al., 2013) for participants *Pi*. Dummy coding was used with simple addition word problems serving as the reference category. Accordingly, the factor level of *difficulty* was 0 for simple (i.e., non-carry/non-borrow) word problems and 1 for complex (i.e., carry/borrow) word problems, and the factor of *operation* was 0 for addition word problems and 1 for subtraction word problems. *β*_0_ reflects the mean for simple addition (i.e., estimate for reference category), *β*_1_ reflects the difference between simple addition and complex addition (i.e., estimate for main effect of difficulty), *β*_2_ reflects the difference between simple addition and simple subtraction (i.e., estimate for main effect of operation), and *β*_3_ reflects the overadditive difference between simple addition and complex subtraction on top of the two main effects (i.e., estimate for interaction effect of difficulty and operation). The error term was named *ε*_*ij*_.

After fitting the LMM, both fixed and random effects were tested for significance by applying the function *step* from the R-package *lmerTest*, which performs automatic backward LMM selection with F-tests using Satterthwaite’s method to estimate degrees of freedom. This function tests random effects first and fixed effects in a hierarchical order afterwards. In case of the current models, random effects *S*_*Pi*_ were tested first, the interaction term *β*_3_ was tested next, and main effects *β*_1_ and *β*_2_ were tested last. When a term was non-significant, the model was re-estimated without the respective parameters for the next step.

## Results

### Data preprocessing

All 63 participants completed the entire study consisting of 32 word problems, so that all datasets were included in the data analysis. There were no missing responses. Incorrect responses led to exclusion of 469 out of 2016 trials (i.e., 23.26%) for the analysis of FD, FC, RD, RC, NN, TT, TN and RT (but not of ER). More precisely, 353 out of 1008 non-solvable trials (i.e., 35.02%) and 116 out of 1008 solvable trials (i.e., 11.51%) were incorrectly answered and therefore excluded. As shown in Supplementary Material H, we looked at accuracies for all word problems separately and checked whether any of the eight stories was frequently misunderstood and thus incorrectly responded to in more than 33% of the trials either within the two solvable or within the two non-solvable versions. This was the case for the “Cinema” story, where only 7.14% of the non-solvable versions were answered (see Table H1), so that all four “Cinema” word problems were excluded from all further analyses. For a better overview of all dependent variables (FD, FC, RD, RC, NN, TT, TN, RT, and ER) for each participant and word problem, we illustrated the means for each combination of solvability and operation (see Supplementary Material I). Note that difficulty (carry/borrow and non-carry/non-borrow) was counterbalanced between but not within these combinations.

### Static eye-tracking measures

**Fig. 1:**
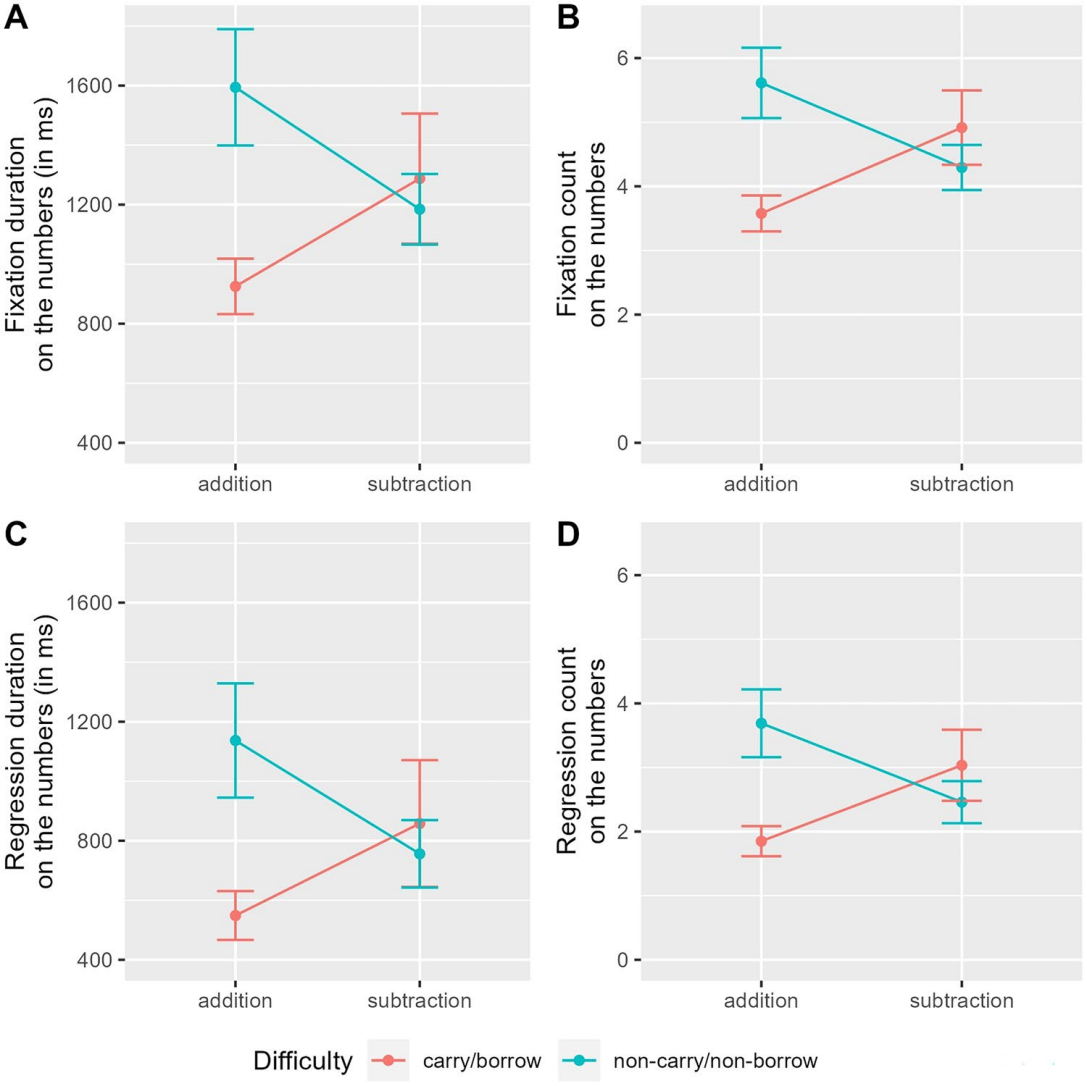
This figure illustrates **A** FD in milliseconds, **B** FC, **C** RD in milliseconds, and **D** RC on the numbers (AOI) in non-solvable word problems. The mean is plotted for each operation (addition vs. subtraction) depending on difficulty (blue: non-carry/non-borrow; red: carry/borrow) with error bars indicating plus/minus one standard error

The pattern of all static eye-tracking measures (FD, FC, RD, and RC) for the numbers (AOI) in correctly answered non-solvable word problems was similar. In all four variables, a significant interaction effect revealed that, as compared to the switch from addition to subtraction and from simple to complex, there was an overadditive effect when switching from simple addition to complex subtraction (see Tables 4, 5, 6, and 7). This crossover interaction is illustrated in Fig. 1. Moreover, the baseline of all static eye-tracking measures varied between participants, as reflected by significant random intercepts in all four LMMs. Because of the significant interaction effect, lower-level effects (i.e., main effects operation and difficulty) were not tested but remained in the LMM. As compared to the reference category of simple addition problems, descriptively less attention was drawn to simple subtraction and complex addition problems. Note that the results are similar for all four static eye-tracking variables, because FD, FC, RD, and RC are not independent from one another.

**Table 4:** LMM results for FD (in milliseconds) on the numbers (AOI) within correctly answered non-solvable word problems

Parameter	Meaning	Estimate	F-test/LRT	p-value
*β*_0_	Fixed: intercept_NCNB-Add_	1608.74	–	–
*S*_0*Pi*_	Random: participant	457.76	23.29	< 0.001*
*β*_*1*_	Fixed: difficulty_CB_	−643.60	–	–
*β*_*2*_	Fixed: operation_Sub_	−394.97	–	–
*β*_*3*_	Fixed: interaction_CB-Sub_	734.57	15.02	< 0.001*

The parameter estimates refer to the LMM equation explained in the paragraph “Data analysis” of the “Method” section. Dummy-coding was used, with the fixed intercept reflecting the estimate for non-carry addition word problems and serving as reference category. Parameter estimates for other factor levels revealing significant fixed effects refer to differences between the reference category and the respective factor level (Add = addition, Sub = subtraction, NCNB = non-carry/non-borrow, CB = carry/borrow). For fixed effects (β), the F-test statistic is displayed, whereas for random effects (S), the Likelihood-Ratio-Test (LRT) statistic is displayed. Estimates for random effects (i.e., intercept variabilities between participants P and word problems W) are standard deviations. The last column shows the p-value corresponding to the F-test or to the LRT, with an asterisk for significant predictors of FD.

**Table 5:** LMM results for FC on the numbers (AOI) within correctly answered non-solvable word problems (for further explanations, see description of Table 4)

Parameter	Meaning	Estimate	F-test/LRT	p-value
*β*_0_	Fixed: intercept_NCNB-Add_	5.66	–	–
*S*_0*Pi*_	Random: participant	1.51	40.99	< 0.001*
*β*_*1*_	Fixed: difficulty_CB_	−1.96	–	–
*β*_*2*_	Fixed: operation_Sub_	−1.25	–	–
*β*_*3*_	Fixed: interaction_CB-Sub_	2.56	24.52	< 0.001*

**Table 6:** LMM results for RD (in milliseconds) on the numbers (AOI) within correctly answered non-solvable word problems (for further explanations, see description of Table 4)

Parameter	Meaning	Estimate	F-test/LRT	p-value
*β*_0_	Fixed: intercept_NCNB-Add_	1148.60	–	–
*S*_0*Pi*_	Random: participant	427.02	20.23	< 0.001*
*β*_*1*_	Fixed: difficulty_CB_	−563.93	–	–
*β*_*2*_	Fixed: operation_Sub_	−367.25	–	–
*β*_*3*_	Fixed: interaction_CB-Sub_	657.45	12.79	< 0.001*

**Table 7:** LMM results for RC on the numbers (AOI) within correctly answered non-solvable word problems (for further explanations, see description of Table (4)

Parameter	Meaning	Estimate	F-test/LRT	p-value
*β*_0_	Fixed: intercept_NCNB-Add_	3.72	–	–
*S*_0*Pi*_	Random: participant	1.35	33.70	< 0.001*
*β*_*1*_	Fixed: difficulty_CB_	−1.77	–	–
*β*_*2*_	Fixed: operation_Sub_	−1.18	–	–
*β*_*3*_	Fixed: interaction_CB-Sub_	2.33	22.53	< 0.001*

### Dynamic eye-tracking measures

**Fig. 2:**
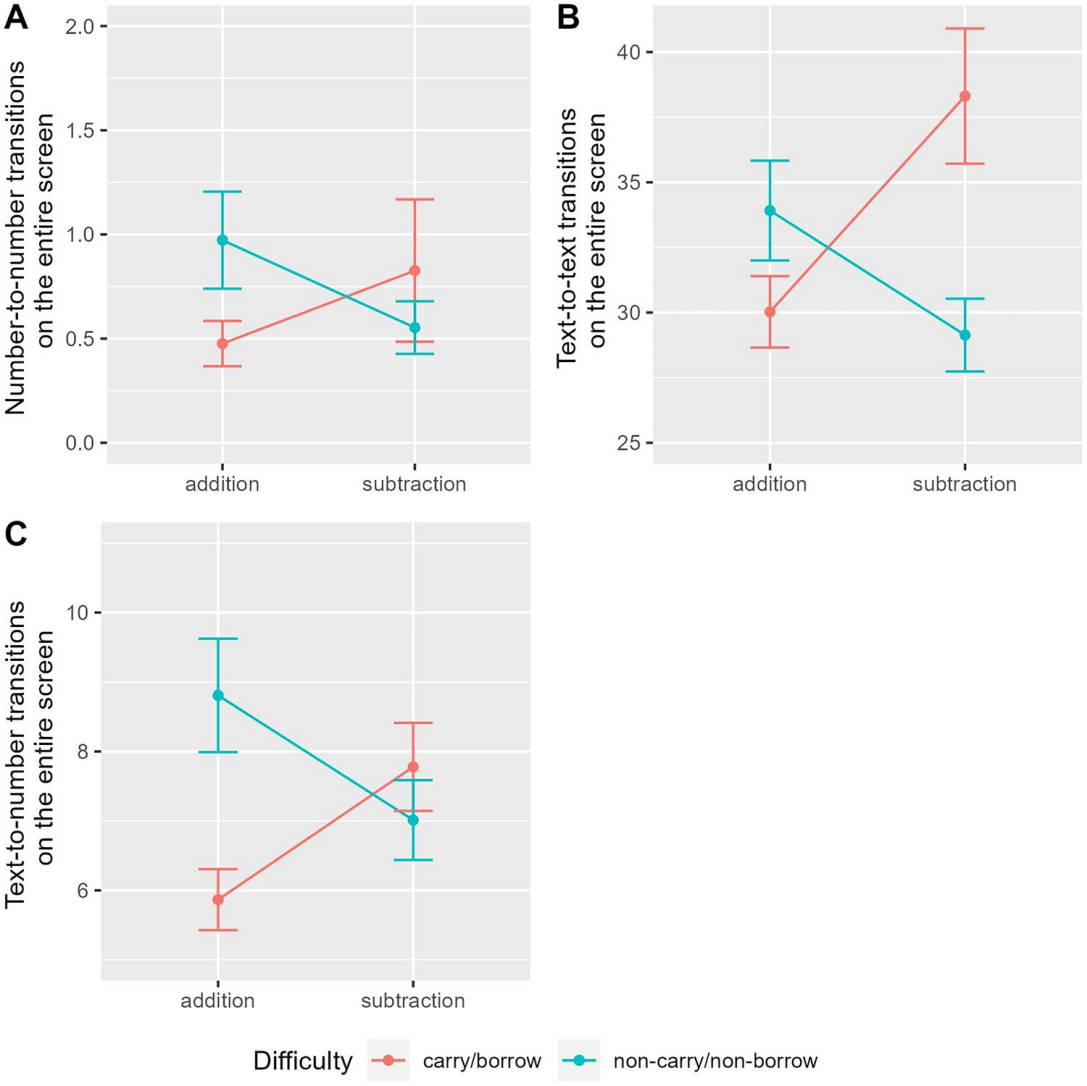
This figure illustrates **A** NN transitions, **B** TT transitions, and **C** TN transitions in non-solvable word problems. The mean is plotted for each operation (addition vs. subtraction) depending on difficulty (blue: non-carry/non-borrow; red: carry/borrow) with error bars indicating plus/minus one standard error

The pattern of all dynamic eye-tracking measures (NN, TT, and TN) in correctly answered non-solvable word problems was similar to the pattern of all static eye-tracking measures (FD, FC, RD, and RC). In all three variables, a significant interaction effect revealed that, as compared to the switch from addition to subtraction and from simple to complex, there was an overadditive effect when switching from simple addition to complex subtraction (see Tables 8, 9, and 10). This crossover interaction is illustrated in Fig. 2. Moreover, the baseline of all dynamic eye-tracking measures varied between participants, as reflected by significant random intercepts in all three LMMs. Because of the significant interaction effect, lower-level effects (i.e., main effects operation and difficulty) were not tested but remained in the LMM. As compared to the reference category of simple addition problems, descriptively less transitions were made in simple subtraction and complex addition problems. Note that, opposed to the four static eye-tracking variables, the results for the three dynamic eye-tracking variables are independent from one another.

**Table 8:** LMM results for NN transition counts within correctly answered non-solvable word problems (for further explanations, see description of Table 4)

Parameter	Meaning	Estimate	F-test/LRT	p-value
*β*_0_	Fixed: intercept_NCNB-Add_	0.98	–	–
*S*_0*Pi*_	Random: participant	0.48	8.68	0.003*
*β*_*1*_	Fixed: difficulty_CB_	−0.48	–	–
*β*_*2*_	Fixed: operation_Sub_	−0.40	–	–
*β*_*3*_	Fixed: interaction_CB-Sub_	0.75	8.25	0.004*

**Table 9:** LMM results for TT transition counts within correctly answered non-solvable word problems (for further explanations, see description of Table 4)

Parameter	Meaning	Estimate	F-test/LRT	p-value
*β*_0_	Fixed: intercept_NCNB-Add_	34.53	–	–
*S*_0*Pi*_	Random: participant	7.64	91.07	< 0.001*
*β*_*1*_	Fixed: difficulty_CB_	−4.03	–	–
*β*_*2*_	Fixed: operation_Sub_	−5.01	–	–
*β*_*3*_	Fixed: interaction_CB-Sub_	13.51	44.82	< 0.001*

**Table 10:** LMM results for TN transition counts within correctly answered non-solvable word problems (for further explanations, see description of Table 4)

Parameter	Meaning	Estimate	F-test/LRT	p-value
*β*_0_	Fixed: intercept_NCNB-Add_	8.86	–	–
*S*_0*Pi*_	Random: participant	2.11	45.64	< 0.001*
*β*_*1*_	Fixed: difficulty_CB_	−2.82	–	–
*β*_*2*_	Fixed: operation_Sub_	−1.71	–	–
*β*_*3*_	Fixed: interaction_CB-Sub_	3.57	25.05	< 0.001*

### Performance measures

**Fig. 3:**
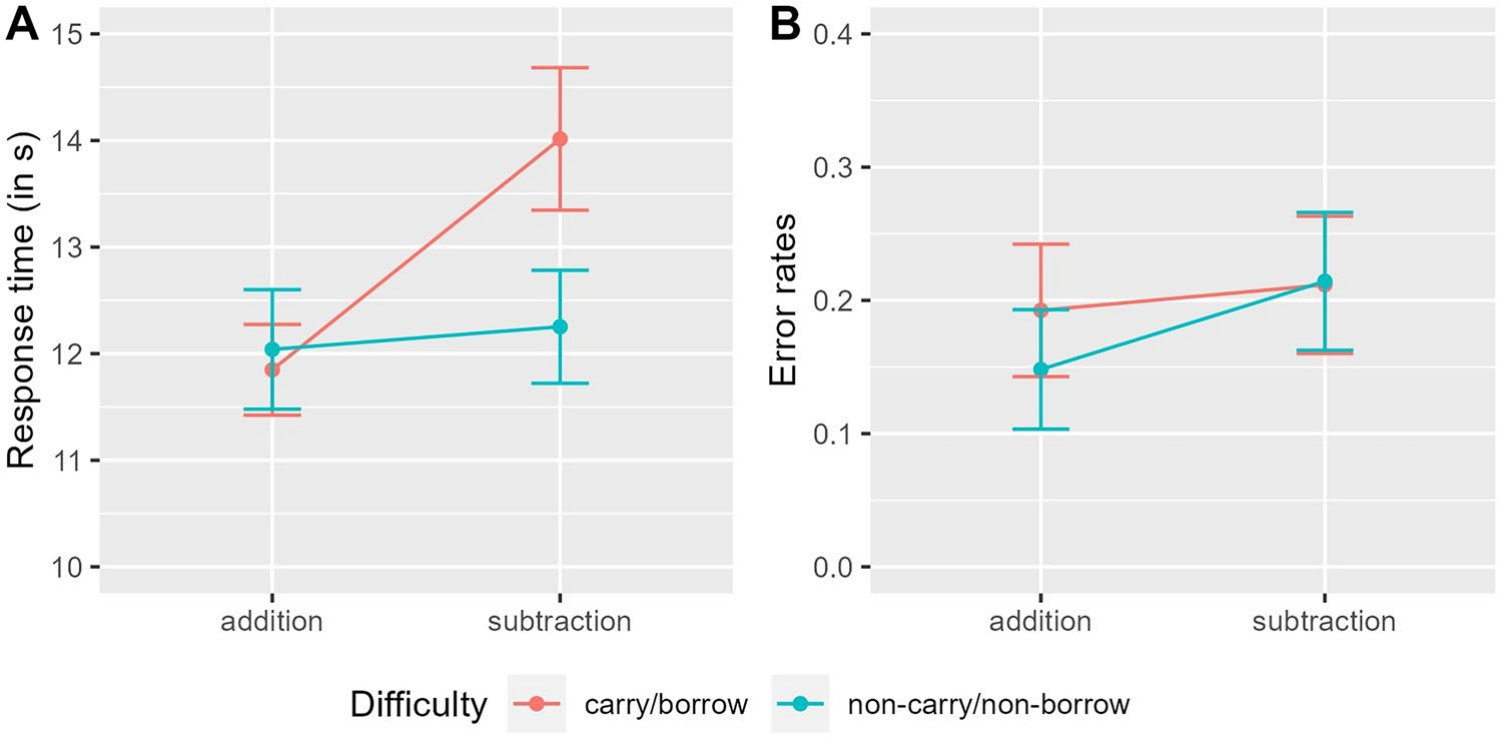
This figure illustrates **A** RT in seconds for correctly answered non-solvable word problems, and **B** ER for all non-solvable word problems. The mean is plotted for each operation (addition vs. subtraction) depending on difficulty (blue: non-carry/non-borrow; red: carry/borrow) with error bars indicating plus/minus one standard error

A significant interaction effect between operation and difficulty was found for RT in correctly answered non-solvable word problems. This effect revealed that, as compared to the switch from addition to subtraction and from simple to complex, participants responded overadditively slower to complex subtraction than to simple addition problems (see Table 11). Moreover, the baseline of RT varied between participants, as reflected by significant random intercepts in the LMM. Because of the significant interaction effect, lower-level effects (i.e., main effects operation and difficulty) were not tested but remained in the LMM. Responses were descriptively slower in non-solvable simple subtraction than simple addition word problems. Further, responses were descriptively faster in non-solvable complex addition than simple addition word problems. RT are illustrated in Fig. 3A.

**Table 11:** LMM results for RT (in seconds) within correctly answered non-solvable word problems (for further explanations, see description of Table 4)

Parameter	Meaning	Estimate	F-test/LRT	p-value
*β*_0_	Fixed: intercept_NCNB-Add_	12.09	–	–
*S*_0*Pi*_	Random: participant	2.27	313.66	< 0.001*
*β*_*1*_	Fixed: difficulty_CB_	−0.19	–	–
*β*_*2*_	Fixed: operation_Sub_	0.19	–	–
*β*_*3*_	Fixed: interaction_CB-Sub_	2.03	26.49	< 0.001*

No significant interaction between operation and difficulty was detected for ER in non-solvable word problems. However, a significant main effect of operation revealed that significantly more errors were made in non-solvable subtraction than addition word problems (see Table 12). Moreover, the baseline of ER varied between participants, as reflected by significant random intercepts in the LMM. ER are illustrated in Fig. 3B.

**Table 12:** LMM results for ER within non-solvable word problems (for further explanations, see description of Table 4)

Parameter	Meaning	Estimate	F-test/LRT	p-value
*β*_0_	Fixed: intercept_NCNB-Add_	0.17	–	–
*S*_0*Pi*_	Random: participant	0.09	30.06	< 0.001*
*β*_*1*_	Fixed: difficulty_CB_	–	1.26	0.261
*β*_*2*_	Fixed: operation_Sub_	0.04	4.68	0.031*
*β*_*3*_	Fixed: interaction_CB-Sub_	–	1.60	0.205

## Discussion

This study investigated the nature of number processing (including both basic steps, such as recognizing numbers and understanding their magnitude, and late steps, such as performing calculations) and its timing in relation to text processing within solvable and non-solvable arithmetic word problems. We explored whether text and number processing occur in sequential steps or whether they interact, and whether numbers are processed as early as the text. To delve into this question, we examined not only behavioral measures but also eye-tracking measures. Specifically, we analyzed both static eye-tracking measures (i.e., fixations and regressions) and dynamic eye movements (i.e., transitions) between text and numbers. Importantly, in this study, both solvable and non-solvable arithmetic word problems were used, with their level of numerical difficulty being manipulated by the need for a carry/borrow operation.

### Number processing in word problems

In the present study, both behavioral and eye-tracking measures suggest simultaneous number and text processing. However, as revealed by an interaction of difficulty (carry/borrow vs. non-carry/non-borrow) and operation (addition vs. subtraction), the effectiveness of this common processing strategy depends on the type of problem, indicating that different strategies may be employed in various contexts. Please note that in all measures, interindividual variability influenced the results, as indicated by significant random participant intercepts.

#### Behavioral measures and number processing

In non-solvable word problems, a significant interaction between difficulty and operation was found. Specifically, borrow subtraction word problems presented a unique challenge to participants, leading to noticeably longer RT compared to the three remaining problem types. In other words, the data pointed towards a borrow effect, but not a carry effect (see Fig. 3A). This observation supports the hypothesis that number processing is activated early on in non-solvable word problems. It cannot be located sequentially after text processing, because in correctly solved non-solvable problems, no arithmetic processing would take place according to sequential models. Therefore, any effect of arithmetic difficulty manipulation in non-solvable word problems is inconsistent with strictly sequential models.

Regarding ER, a main effect was observed for the type of operation, but not for the need of carry/borrow. Subtraction problems elicited a higher ER, whereas the need of carry/borrow did not have a significant impact. The increased ER associated with subtraction also seems to suggest simultaneous text and number processing. Subtraction might be particularly linked to the problem-solving phase where the mental representation of the problem is constructed, as shown in the study by Daroczy et al. (2023). Contrary to other studies, we did not detect direct evidence for carry/borrow effects. For instance, Dresen et al. (2020) found a clear carry/borrow effect in word problems, which was replicated by Daroczy et al. (2023); however, in both studies, the used problems were solvable and required a calculation. This discrepancy either suggests that numbers were not fully processed in non-solvable problems, so that no carry/borrow effects existed, or that these effects are so weak that the statistical power to detect the carry/borrow effect was not high enough.

In summary, however, the behavioral results already indicate that number processing occurs much earlier than assumed in some influential models. Specifically, the present findings indicate that even in non-solvable word problems, hypothetical carrying or borrowing reduces performance, and subtraction is perceived as more difficult than addition, although the results do not offer a complete picture. Note that in solvable word problems, both main effects were significant for both performance measures (see Supplementary Material F). That is, RT were longer and ER higher in subtraction than in addition word problems and in carry/borrow than in non-carry/non-borrow word problems. Based on these outcomes, number processing, especially potential calculation, appears to play a role even in non-solvable word problems (i.e., not only in solvable word problems). Eye-tracking data could uncover very nuanced effects here.

#### Static eye tracking measures and number processing

According to Park et al. (2015) and Mason et al. (2016), more eye movements indicate a higher level of interactive processing between text and numbers. Consistent with this finding, for all four static dependent variables (i.e., FD, FC, RD, and RC on numbers), a disordinal interaction presenting a very clear pattern between difficulty and operation was identified (see Fig. 1). Descriptively, less attention was drawn to numbers in difficult than in easy addition problems, as reflected by shorter and less numerous fixations and regressions on numbers. In word problems involving addition, it seems that when calculations are straightforward, individuals tend to initiate the calculation process, as reflected by them looking at the numbers longer and more frequently. In contrast, when it would be more difficult to calculate with the numbers, individuals seem to (consciously or unconsciously) make the decision not to calculate and first check the solvability of the word problems. Interestingly, the pattern was reversed for subtraction problems, where less attention was drawn to numbers in easy than in difficult subtraction problems. Note that in solvable word problems, the interaction turned out to be significant for all static dependent variables as well (see Supplementary Material D). In contrast to the disordinal interaction observed in non-solvable word problems, the interaction seemed to be ordinal in solvable word problems with an overadditive difficulty effect for borrow subtraction problems. In sum, while static eye-tracking measures in subtraction problems increased when borrowing was required, they decreased in addition problems when carrying was required. The borrow effect in subtraction was observed as expected both in solvable and non-solvable problems in the current study, but the observed reversed carry effect in addition was surprising and deserves some attention. Before we discuss this matter, we elaborate on the pattern observed in the dynamic eye-tracking measures, which exhibit a similar, equally surprising trend.

#### Dynamic eye-tracking measures and number processing

The patterns observed in dynamic eye-tracking measures mirrored those observed in static eye-tracking measures, indicating consistency regarding the operationalization of cognitive processing using different measurement modalities. In NN and TN transitions (see Fig. 2A, C), the patterns are similar to the disordinal interaction of the static measures (see Fig. 1). Namely, in addition problems with the need to carry, the number of transitions was descriptively lowest, whereas in addition problems without the need to carry, the number of transitions was highest. In TT transitions, the pattern looks slightly different (see Fig. 2B), such that descriptively most TT transitions were made in subtraction problems when borrowing was required, whereas least were made in addition problems when no carrying was required. Please note that there are a lot more TT and TN than NN transitions. The reported increased number of transitions is thus primarily associated with heightened interactive attention between text parts and between numbers and text. Therefore, it is possible that simple, non-solvable problems require a more comprehensive integration of text and numbers. In solvable word problems, the main effects of both difficulty and operation were significant for NN, while only the main effect of operation was significant for TN and no significant effect was found for TT (see Supplementary Material E). However, in contrast to non-solvable word problems, the interaction did not reach significance for any of the three dynamic eye-tracking measures.

In sum, the results speak for interactive processing of text and numbers. Indeed, numbers play a significant role in word problem solving and are processed even when they are not needed. We will explore the possible underlying mechanisms in greater detail in the following paragraph, aiming to elucidate how these interactions influence problem solving.

### Interaction of text and number processing in word problem solving models

The disordinal interaction between operation and difficulty in arithmetic word problems reflects that numbers start to be processed before the text is fully processed and before it is understood whether the problem is solvable or non-solvable. This suggests an integration of number and text processing, and it is possible that even more processes are involved simultaneously in word-problem solving. Our findings are supported by Zhou et al. (2018), who found evidence in neural correlates that, contrary to the view of a dissociation of text and number processing as shown by neuroimaging studies, the dissociation does not imply a complete separation of number processing from text processing due to the involvement of the semantic system. Nevertheless, these studies did not investigate the neural correlates of the carry/borrow effect within word problems. Our results provide compelling evidence for a magnitude-based mental representation, as supported by Bergqvist and Österholm (2010), whose model incorporates a cyclic component that enables it to update mental representations. Moreover, our findings are in line with conclusions drawn from pure text comprehension studies (without numerical information and mathematical operations to be performed), claiming that mental representations are built up in an incremental manner, allowing for regular updates during the reading process (Gernsbacher et al., 1998). This suggests that the mental representation of word problems is delicate, using an economical and effective parallel method for managing cognitive resources. This finding provides evidence against the sequential model proposed by Kintsch and Greeno (1985), who argue for a separation of text and number processing. According to their sequential model, a mental representation of a word problem is built by processing the surface and understanding the text semantics, and, in the next step, extending this text model by a problem model to select a fitting schema, fill it in with numbers, and finally carry out the calculation. According to this model, simply reading a non-solvable word problem should be sufficient to recognize that it is non-solvable, and a calculation should not even be initiated if the text of a non-solvable word problem is correctly understood. Notably, the significant interaction between difficulty and operation in non-solvable problems highlights that number manipulation influenced the solving process, which would not be the case if participants first read the text before initiating a calculation. Note that to rule out potential influences of the exact text and used story, all problems were standardized for word count, letter count, and word frequency (Roth, 2021). Thus, although the exact text and used story was counterbalanced within solvability and operation but not within difficulty (see Supplementary Materials A and I), it is highly unlikely that the observed interaction is due to textual differences.

Other models which try to explain how word problems are solved account for numbers in various ways but do not fully clarify what occurs in certain scenarios. The schema model and the situation model suggest that numbers are not necessarily processed from the beginning of the solving process. The SECO model examines how numbers function within word problems, attributing importance to the role and meaning of numbers as they semantically relate to the mental representation in word problems. The semantic relatedness was intentionally omitted from our study because the research aimed to explore number processing in a more abstract sense, specifically how arithmetic operations like carrying or borrowing influence problem-solving, without considering the contextual or semantic significance of the numbers involved. Our results suggest a more generalized role for numbers in problem-solving, indicating that numbers may play an early role in cognitive processing, even when they are not directly connected to semantic meanings.

To explain the disordinality of the observed interaction, we propose that the solving process is relatively adaptable and can shift based on the perceived or actual difficulty of the problems. Following the model proposed by Bergqvist and Österholm (2010), the initial reading (possibly skimming) of a non-solvable problem leads to a mental representation of the content. Based on this mental representation, a suitable strategy is chosen: re-reading, calculating, or ceasing the solving process. In some cases, individuals use the initial mental representation to label the problem as “solvable” and calculate or as “non-solvable” and cease the solving process, without re-reading the text. Such processes are supported by the fact that some behavioral carry or borrow effects are weaker than in previous studies. However, in other cases, individuals may opt to re-read the text, which might result in a modification of their initial mental representation (i.e., from a solvable to a non-solvable representation). In such cases, an extra step is necessary to correctly construct a mental representationand identify simple non-solvable problems as “non-solvable.” This additional step necessitates resources, which manifest as fixations, regressions, and transitions. Crucially, this process seems to be moderated by the problem’s perceived difficulty, aligning with the findings by Doz et al. (2023) and speaking against mutually exclusive strategies and instead for calculation already happening during reading and re-reading, most likely depending on the perceived difficulty of the problems.

The above reasoning explains well why there might be a borrow effect (as reflected by more fixations, regressions, and transitions) in non-solvable subtraction word problems. Namely, when a problem is non-solvable and involves borrowing, individuals might need more cognitive resources to confirm its non-solvability. Following the same reasoning, one would expect a similar pattern in addition problems, specifically a carry effect in non-solvable addition word problems. However, the observed crossover interaction reveals a descriptively reversed trend in addition, such that less attention is drawn to simple (non-carry) than to difficult (carry) non-solvable addition problems. One possible explanation is that individuals employ a direct translation strategy (e.g., Hegarty et al., 1995) when they perceive both the text and numbers as easy and initiate a calculation as it requires minimal cognitive resources (i.e., in the case of the non-carry addition problems, representing the easiest of the four problem types in the current study). They might then revise their problem representationor abandon it, depending on the complexity of the problem, which is defined as the combination of factors resulting from carry/non-carry in combination with addition/subtraction, along with other factors such as solvability and non-solvability. Nonetheless, even in case of abandonment of the calculation, a mismatch with the initial mental representationis recognized, indicating at least partial integration of number processing and text processing. The decision of whether to calculate may be influenced not only by motivational aspects but also by the simple act of “giving up” (Bergqvist & Österholm, 2010). Specifically, on a response (decision) level, participants may lower their threshold for answering that the presented word problem is non-solvable if the potential calculation is deemed as relatively complicated. In this case, it would be more likely and faster that participants abandon the calculation and respond that the problem is non-solvable. This would lead to less eye movements on the numbers in difficult than in easy non-solvable word problems. This notion is supported by the fact that the need for carrying is usually recognized relatively early during the encoding of the problem (Moeller et al., 2011b), so individuals could choose to capitulate relatively early.

While this explanation holds well for the eye-tracking data from addition word problems and the carry effect, it is, however, important to note that this explanation does not account for all findings of the current study. Namely, it does not account for the results of subtraction word problems and the borrow effect, where the more difficult arithmetic condition leads to more eye-movements and no such tendencies to abandon calculation or “giving up” can be observed. While both explanations (i.e., accounting for the addition findings and the carry effect on the one hand, and accounting for the subtraction findings and the borrow effect on the other hand) are possible and in line with some previous theoretical assumptions, we have no convincing explanation of why one explanation seems to be true for addition and the other for subtraction.

A different explanation for the reversed carry effect in static measures (i.e., fixations and regressions) for addition problems as reflected by the significant interactions might be addition is the default operation for many participants. That is, when faced with two numbers and before knowing what operation is actually required, addition might be more likely to be performed as compared to subtraction or other arithmetic operations. This assumption can be derived from, on the one hand, participants getting more involved in simple addition word problems than in complex ones (as reflected by more fixations and regressions in non-carry problems than in carry problems), but, on the other hand, no stronger involvement in simple subtraction word problems than in complex ones. Importantly, if an addition was performed with the two numbers in the subtraction problems, half of them would be carry problems and half of them would be non-carry problems. Thus, addition as a default operation would explain that there is on average no difference between borrow problems and non-borrow problems.

Importantly, it also remains unclear whether difficulty had an effect apart from its interaction with operation. As the interaction was significant and describes the eye movement patterns more precisely, the main effects of difficulty and operation were not even tested within the LMMs for any of the four measures. The descriptive pattern of results is consistent with the explanatory approach developed above. Another argument that supports this idea is that the average RT for solvable problems, where calculation is necessary to provide a correct solution, is much higher than for non-solvable problems, where mere reading comprehension is enough to answer correctly. Overall, it can be concluded that there is evidence for interactive processing of text and numbers and for different strategies in addition and subtraction word problems. Indeed, numbers play a significant role in word problem solving and are processed even when not required. These arguments are supported by Orrantina and Múñez(2013), who found suggesting that an automatic, analog magnitude-based mental representation is routinely activated during word problem solving. Our research not only supports these findings but provides even stronger evidence, as we even manipulated the calculation within the word problems.

### Limitations and future research

Although the current study could clearly demonstrate that number processing takes part at an early stage in word problem solving, there are some limitations and future research questions to be considered. Firstly, the study employed a 2 (solvability) * 2 (operation) * 2 (difficulty) design, leading to eight word-problem types. Importantly, we decided to use each story for no more than four word problems. The reason for this was that if each story would have been repeated eight times, participants might have ceased to engage with the text thoroughly, potentially skipping most of the reading at some point due to familiarity, learning effects, and efficiency concerns. Thus, the design was not fully counterbalanced, but only across difficulty (carry/borrow), but only across solvability and operation. To mitigate the effects of this unbalanced design, we controlled for linguistic difficulty across the word problems. Therefore, we can argue that any differences due to linguistic difficulty arising from mean word frequencies, average sentence length, number of words, and unequal use of stories are absent or negligible. However, there might be other, not yet considered (or even unknown), factors that make word problems more difficult in certain conditions than in others.

Secondly, relational terms such as “more” or “less” should not be overlooked, as they are keywords for the arithmetic operations addition and subtraction, respectively. The evidence of interactive processing underscores the importance of closely examining integrative attention between text and numbers. Moreover, in several word problems, the second sentence already pointed towards an arithmetic operation (e.g., the words “in total” in the simple solvable “Museum” problem or in the complex solvable “Gardening” problem pointed towards subtraction), whereas in most other problems, this cue appeared only in the third sentence. Future studies should ensure the placement of keywords pointing towards a specific mathematical operation is consistent across word problems or is manipulated as a factor.

Thirdly, some used word problems in this study might have seemed ambiguous in terms of whether they were solvable or non-solvable. For instance, the simple non-solvable “Cinema” problem contained the information that two classes of 41 and 27 children and their teachers went to the cinema and asked about how many seats they needed. Since the number of teachers joining the trip to the cinema was not mentioned, the word problem was non-solvable, but some participants might have assumed that one teacher joined per class. This might explain why only 7.14% of the two (simple and complex) non-solvable versions of the “Cinema” word problems were identified as non-solvable. Moreover, in the non-solvable versions of the “Tennis,” “Museum,” “Busses,” and “Party” problems, some participants might have executed a calculation to find a minimum or maximum as an answer. For instance, if Sophie invites 24 and Mia invites 18 friends to their party, assuming that none of their friends declines the invitation, the maximum of 42 friends would come. The “Marbles,” “Gardening,” and “Fruits” problems, in contrast, might have been less ambiguous and thus more suitably constructed for the present investigation focusing on non-solvable word problems. In addition, cultural context might impact the perceived solvability of problems. Participants’ approaches to problem-solving and their assumptions about a problem’s solvability can be shaped by cultural norms and experiences, which influence their cognitive processing strategies (e.g., Rhodes et al., 2024). Therefore, we recommend that word problems should be specifically selected and tailored to fit cultural contexts.

Forthly, we should note that fixations and regressions of AOI, as well as transitions between parts of the text and numbers, do not capture the full calculation process. Rottmann and Schipper (2002) demonstrated that children tend to shift their gaze upwards or to an imaginary point when concentrating, which is potentially not even on the computer screen and therefore not recorded in eye-tracking. It is plausible that fixations, regressions, and transitions outside of AOI also indicate important cognitive processes in adults. Thus, a subsequent study could capture eye movements that do not target the word problem but instead points that are not located on the computer screen.

Lastly, we acknowledge that whether the question is fully read and whether attention is paid to the question early or late during the solving process may indeed play a crucial role in the word problem-solving process. However, to fully explore this aspect, we recommend conducting experiments specifically designed to investigate the unique role of the question within the processing of word problems. In general, our study does not allow to exactly locate when and how calculation and other processes take place and interact. We encourage other researchers to explore this question further by designing studies that can better isolate and identify processes and their interactions. Additionally, we hope that such future work will refine the existing theoretical framework.

### Theoretical and practical implications

The theoretical implication that calculation, or at least some form of number processing in word problems, occurs in parallel with text processing is profound. It challenges traditional models that suggest a sequential approach, where text processing (including reading comprehension) and number processing (including potential calculation) are distinct, subsequent phases. This finding supports a more integrated model, where number processing and text processing are not discrete, but interwoven from the outset. Ultimately, we require a new theoretical model that considers that number processing, including calculation steps, is a dynamic component of the word problem-solving process which varies according to the type of problem (i.e., solvability, operation, and arithmetic difficulty) and potentially its formulation as well.

## Conclusion

Overall, our findings contribute to a deeper understanding of the cognitive processes involved in solving non-solvable word problems and underscore the importance of considering both individual- and problem-related factors in educational and research contexts. Firstly, the study results suggest that attention is already directed towards the numbers while reading a arithmetic word problem. A strict separation of text and number processing does not seem appropriate in view of the present results. Secondly, solution strategies seem to depend on the exact problem type and are more fragile and flexible as previously assumed. By elucidating the nuanced interplay between mathematical operations, problem difficulty, and cognitive processing, our study provides valuable insights about the complexity of word-problem solving.

## Supplementary Information

Below is the link to the electronic supplementary material.

Supplementary file1 (DOCX 3384 KB)

## Data Availability

The anonymized datasets and all R scripts for the data preprocessing and analysis can be found on the Open Science Framework (https://osf.io/nvdhq/).
