# Supplementary material for: Can’t help processing numbers with text: Eye-tracking evidence for simultaneous instead of sequential processing of text and numbers in arithmetic word problems
Source: Psychol Res. 2025 Jan 20;89(1):50. doi: 10.1007/s00426-024-02069-x (PMC11753309; doi:10.1007/s00426-024-02069-x)

**Supplementary Material A:**

**Stimulus set: Solvable and non-solvable word problems**

This Supplementary Material consists of eight tables that compromise all 32 relevant word problems, categorized by story (A1: Tennis, A2: Marbles, A3: Museum, A4: Party, A5: Gardening, A6: Fruits, A7: Cinema, A8: Busses). As described in the core manuscript, word problems were carefully constructed as parallel versions according to experimental conditions: Each story featured a solvable addition, a solvable subtraction, a non-solvable addition, and a non-solvable subtraction word problem.

**Table A1**

*Four parallel word problems constructed from the Tennis story.*

| Difficulty | Solvability | |
| --- | --- | --- |
|  | solvable | non-solvable |
| simple | addition:  Neuerdings nehmen Paul und Marie Tennis-Training.  *(Lately, Paul and Marie have been taking tennis lessons.)*  Heute hat Paul 62 Bälle getroffen. *(Today, Paul hit 62 balls.)*  Marie hat 31 Bälle mehr getroffen.  *(Marie hit 31 more balls.)*  Wie viele Bälle hat Marie getroffen?  *(How many balls did Marie hit?)* | subtraction:  Neuerdings nehmen Paul und Marie Tennis-Training.  *(Lately, Paul and Marie have been taking tennis lessons.)*  Heute hat Paul 59 Bälle getroffen. *(Today, Paul hit 59 balls.)*  Marie hat 27 Bälle weniger getroffen.  *(Marie hit 27 less balls.)*  Wie viele Bälle hat der Trainer getroffen?  *(How many balls did the trainer hit?)* |
| complex | subtraction:  Neuerdings nehmen Paul und Marie Tennis-Training.  *(Lately, Paul and Marie have been taking tennis lessons.)*  Heute hat Paul 43 Bälle getroffen. *(Today, Paul hit 43 balls.)*  Marie hat 16 Bälle weniger getroffen.  *(Marie hit 16 less balls.)*  Wie viele Bälle hat Marie getroffen?  *(How many balls did Marie hit?)* | addition:  Neuerdings nehmen Paul und Marie Tennis-Training.  *(Lately, Paul and Marie have been taking tennis lessons.)*  Heute hat Paul 69 Bälle getroffen.  *(Today, Paul hit 69 balls.)*  Marie hat 17 Bälle mehr getroffen.  *(Marie hit 17 more balls.)*  Wie viele Bälle hat der Trainer getroffen?  *(How many balls did the trainer hit?)* |

**Table A2**

*Four parallel word problems constructed from the Marbles story.*

| Difficulty | Solvability | |
| --- | --- | --- |
|  | solvable | non-solvable |
| simple | subtraction:  Max und Leon spielen mit Murmeln.  *(Max and Leon are playing with marbles.)*  Max besitzt 59 Murmeln.  *(Max owns 59 marbles.)*  Er hat 25 mehr als Leon.  *(He has 25 more than Leon.)*  Wie viele Murmeln hat Leon?  *(How many marbles does Leon have?)* | addition:  Max und Leon spielen mit Murmeln.  *(Max and Leon are playing with marbles.)*  Max besitzt 72 Murmeln.  *(Max owns 72 marbles.)*  Er hat 26 weniger als Leon.  *(He has 26 less than Leon.)*  Wie viele Murmeln gibt er Leon?  *(How many marbles does he give to Leon?)* |
| complex | addition:  Max und Leon spielen mit Murmeln.  *(Max and Leon are playing with marbles.)*  Max besitzt 67 Murmeln.  *(Max owns 67 marbles.)*  Er hat 26 weniger als Leon.  *(He has 26 less than Leon.)*  Wie viele Murmeln hat Leon?  *(How many marbles does Leon have?)* | subtraction:  Max und Leon spielen mit Murmeln.  *(Max and Leon are playing with marbles.)*  Max besitzt 56 Murmeln.  *(Max owns 56 marbles.)*  Er hat 27 mehr als Leon.  *(He has 27 more than Leon.)*  Wie viele Murmeln gibt er Leon?  *(How many marbles does he give to Leon?)* |

**Table A3**

*Four parallel word problems constructed from the Museum story.*

| Difficulty | Solvability | |
| --- | --- | --- |
|  | solvable | non-solvable |
| simple | subtraction:  In einem Museum werden Bilder verkauft.  (*Pictures are being sold in a museum.)*  Letztes Wochenende wurden insgesamt 28 Bilder verkauft.  *(Last weekend, 28 pictures were sold in total.)*  Davon wurden 15 Bilder letzten Sonntag verkauft.  *(Of these, 15 pictures were sold last Sunday.)*  Wie viele Bilder wurden letzten Samstag verkauft?  *(How many pictures were sold last Saturday?)* | addition:  In einem Museum werden Bilder verkauft.  *(Pictures are being sold in a museum.)*  Letzten Samstag wurden 25 Bilder verkauft.  *(Last Saturday, 25 pictures were sold.)*  Letzten Sonntag wurden 14 Bilder verkauft.  *(Last Sunday, 14 pictures were sold.)*  Wie viele Bilder waren letztes Wochenende zu verkaufen?  *(How many pictures were for sale last weekend?)* |
| complex | addition:  In einem Museum werden Bilder verkauft.  *(Pictures are being sold in a museum.)*  Letzten Samstag wurden 29 Bilder verkauft.  *(Last Saturday, 29 pictures were sold.)*  Letzten Sonntag wurden 17 Bilder verkauft.  *(Last Sunday, 17 pictures were sold.)*  Wie viele Bilder wurden letztes Wochenende insgesamt verkauft?  *(How many pictures were sold in total last weekend?)* | subtraction:  In einem Museum werden Bilder verkauft.  *(Pictures are being sold in a museum.)*  Letztes Wochenende wurden insgesamt 31 Bilder verkauft.  *(Last weekend, 31 pictures were sold in total.)*  Davon wurden 12 Bilder letzten Sonntag verkauft.  *(Of these, 12 pictures were sold last Sunday.)*  Wie viele Bilder waren letztes Wochenende zu verkaufen?  *(How many pictures were for sale last weekend?)* |

**Table A4**

*Four parallel word problems constructed from the Party story.*

| Difficulty | Solvability | |
| --- | --- | --- |
|  | solvable | non-solvable |
| simple | addition:  Sophie und Mia veranstalten eine Party.  *(Sophie and Mia organize a party.)*  Sophie lädt 52 Freundinnen ein. *(Sophie invites 52 friends.)*  Mia lädt 24 andere Freundinnen ein.  *(Mia invites 24 other friends.)*  Wie viele Freundinnen laden sie insgesamt ein?  *(How many friends do they invite in total?)* | subtraction:  Sophie und Mia veranstalten eine Party.  *(Sophie and Mia organize a party.)*  Dazu laden sie all ihre 38 Freundinnen ein.  *(They invite all their 38 friends.)*  Sophie lädt 24 Freundinnen ein. *(Sophie invites 24 friends.)*  Wie viele Freundinnen kommen zur Party?  *(How many friends are coming to the party?)* |
| complex | subtraction:  Sophie und Mia veranstalten eine Party.  *(Sophie and Mia organize a party.)*  Dazu laden sie all ihre 64 Freundinnen ein.  *(They invite all their 64 friends.)*  Sophie lädt 28 Freundinnen ein.  *(Sophie invites 28 friends.)*  Wie viele andere Freundinnen lädt Mia ein?  *(How many other friends does Mia invite?)* | addition:  Sophie und Mia veranstalten eine Party.  *(Sophie and Mia organize a party.)*  Sophie lädt 24 Freundinnen ein.  *(Sophie invites 24 friends.)*  Mia lädt 18 andere Freundinnen ein.  *(Mia invites 18 other friends.)*  Wie viele Freundinnen kommen zur Party?  *(How many friends are coming to the party?)* |

**Table A5**

*Four parallel word problems constructed from the Gardening story.*

| Difficulty | Solvability | |
| --- | --- | --- |
|  | solvable | non-solvable |
| simple | addition:  Alex beschäftigt sich viel mit Gartenarbeit.  *(Alex spends a lot of time gardening.)*  Letzten Samstag hat er 21 Bäume gepflanzt.  *(Last Saturday, he planted 21 trees.)*  Letzten Sonntag hat er 13 Bäume gepflanzt.  *(Last Sunday, he planted 13 trees.)*  Wie viele Bäume hat er letztes Wochenende insgesamt gepflanzt?  *(How many trees did he plant in total last weekend?)* | addition:  Alex beschäftigt sich viel mit Gartenarbeit.  *(Alex spends a lot of time gardening.)*  Letzten Samstag hat er 32 Bäume gepflanzt.  *(Last Saturday, he planted 32 trees.)*  Letzten Sonntag hat er 13 Bäume gepflanzt.  *(Last Sunday, he planted 13 trees.)*  Wie viele Bäume hat er letzten Freitag gepflanzt?  *(How many trees did he plant last Friday?)* |
| complex | subtraction:  Alex beschäftigt sich viel mit Gartenarbeit.  *(Alex spends a lot of time gardening.)*  Letztes Wochenende hat er insgesamt 37 Bäume gepflanzt.  *(Last weekend, he planted 37 trees in total.)*  Letzten Samstag hat er 19 Bäume gepflanzt.  *(Last Saturday, he planted 19 trees.)*  Wie viele Bäume hat er letzten Sonntag gepflanzt?  *(How many trees did he plant last Sunday?)* | subtraction:  Alex beschäftigt sich viel mit Gartenarbeit.  *(Alex spends a lot of time gardening.)*  Letztes Wochenende hat er insgesamt 34 Bäume gepflanzt.  *(Last weekend, he planted 34 trees in total.)*  Letzten Samstag hat er 15 Bäume gepflanzt.  *(Last Saturday, he planted 15 trees.)*  Wie viele Bäume hat er letzten Freitag gepflanzt?  *(How many trees did he plant last Friday?)* |

**Table A6**

*Four parallel word problems constructed from the Fruits story.*

| Difficulty | Solvability | |
| --- | --- | --- |
|  | solvable | non-solvable |
| simple | subtraction:  Auf unserem Küchentisch steht eine große Obstschale.  *(There is a large fruit bowl on our kitchen table.)*  Darin befinden sich 45 Äpfel.  *(There are 45 apples in it.)*  Außerdem liegen darin 14 Birnen weniger als Äpfel.  *(Besides, there are 14 less pears than apples in it.)*  Wie viele Birnen sind in der Obstschale?  *(How many pears are in the fruit bowl?)* | subtraction:  Auf unserem Küchentisch steht eine große Obstschale.  *(There is a large fruit bowl on our kitchen table.)*  Darin befinden sich 36 Äpfel.  *(There are 36 apples in it.)*  Außerdem liegen darin 13 Birnen weniger als Äpfel.  *(Besides, there are 13 less pears than apples in it.)*  Wie viele Bananen sind in der Obstschale?  *(How many bananas are in the fruit bowl?)* |
| complex | addition:  Auf unserem Küchentisch steht eine große Obstschale.  *(There is a large fruit bowl on our kitchen table.)*  Darin befinden sich 46 Äpfel.  *(There are 46 apples in it.)*  Mama legt 18 Birnen dazu.  *(Mom adds 18 pears to it.)*  Wie viele Stücke Obst sind nun in der Obstschale?  *(How many pieces of fruit are now in the fruit bowl?)* | addition:  Auf unserem Küchentisch steht eine große Obstschale.  *(There is a large fruit bowl on our kitchen table.)*  Darin befinden sich 45 Äpfel.  *(There are 45 apples in it.)*  Mama legt 16 Birnen und einige Bananen dazu.  *(Mom adds 16 pears and some bananas to it.)*  Wie viele Stücke Obst sind nun in der Obstschale?  *(How many pieces of fruit are now in the fruit bowl?)* |

**Table A7**

*Four parallel word problems constructed from the Cinema story.*

| Difficulty | Solvability | |
| --- | --- | --- |
|  | solvable | non-solvable |
| simple | addition:  Zwei Klassen machen einen Ausflug ins Kino.  *(Two classes go on a trip to the cinema.)*  Aus Klasse a kommen 35 Kinder mit.  *(From class a, 35 children are coming on the trip.)*  Aus Klasse b kommen 12 Kinder mit. *(From class b, 12 children are coming on the trip.)*  Wie viele Plätze brauchen die Kinder im Kinosaal?  *(How many seats do they need in the cinema auditorium?)* | addition:  Zwei Klassen und ihre Lehrerinnen machen einen Ausflug ins Kino.  *(Two classes and their teachers go on a trip to the cinema.)*  Aus Klasse a kommen 41 Kinder mit. *(From class a, 41 children are coming on the trip.)*  Aus Klasse b kommen 27 Kinder mit. *(From class b, 27 children are coming on the trip.)*  Wie viele Plätze brauchen sie im Kinosaal?  *(How many seats do they need in the cinema auditorium?)* |
| complex | subtraction:  Zwei Klassen machen einen Ausflug ins Kino.  *(Two classes go on a trip to the cinema.)*  Die Kinder brauchen 34 Plätze im Kinosaal.  *(They need 34 seats in the cinema auditorium.)*  Aus Klasse a kommen 18 Kinder mit.  *(From class a, 18 children are coming on the trip.)*  Wie viele Kinder kommen aus Klasse b mit?  *(How many children are coming on the trip from class b?)* | subtraction:  Zwei Klassen und ihre Lehrerinnen machen einen Ausflug ins Kino.  *(Two classes and their teachers go on a trip to the cinema.)*  Sie brauchen 41 Plätze im Kinosaal. *(They need 41 seats in the cinema auditorium.)*  Aus Klasse a kommen 25 Kinder mit. *(From class a, 25 children are coming on the trip.)*  Wie viele Kinder kommen aus Klasse b mit?  *(How many children are coming on the trip from class b?)* |

**Table A8**

*Four parallel word problems constructed from the Busses story.*

| Difficulty | Solvability | |
| --- | --- | --- |
|  | solvable | non-solvable |
| simple | subtraction:  Alle Kindergartenkinder fahren mit zwei Bussen auf einen Ausflug.  *(All kindergarten children go on a trip on two buses.)*  Insgesamt fahren 38 Kinder mit.  *(A total of 38 children are going.)*  Im ersten Bus sitzen 26 Kinder.  *(There are 26 children sitting on the first bus.)*  Wie viele sitzen im zweiten Bus?  *(How many are sitting on the second bus?)* | subtraction:  Alle Kindergartenkinder fahren mit zwei Bussen auf einen Ausflug.  *(All kindergarten children go on a trip on two buses.)*  Insgesamt fahren 37 Kinder mit.  *(A total of 37 children are going.)*  Im ersten Bus sitzen 16 Kinder.  *(There are 16 children sitting on the first bus.)*  Wie viele Sitze gibt es im zweiten Bus?  *(How many seats are there on the second bus?)* |
| complex | addition:  Alle Kindergartenkinder fahren mit zwei Bussen auf einen Ausflug.  *(All kindergarten children go on a trip on two buses.)*  Im ersten Bus sitzen 32 Kinder.  *(There are 32 children sitting on the first bus.)*  Im zweiten Bus sitzen 29 Kinder. *(There are 29 children sitting on the second bus.)*  Wie viele Kinder fahren insgesamt mit?  *(How many children are going in total?)* | addition:  Alle Kindergartenkinder fahren mit zwei Bussen auf einen Ausflug.  *(All kindergarten children go on a trip on two buses.)*  Im ersten Bus sitzen 28 Kinder.  *(There are 28 children sitting on the first bus.)*  Im zweiten Bus sitzen 19 Kinder.  *(There are 19 children sitting on the second bus.)*  Wie viele Sitze gibt es in den beiden Bussen?  *(How many seats are there on the two buses?)* |

**Supplementary Material B:**

**Stimulus set: Routine and non-routine filler tasks**

This Supplementary Material contains all 16 filler task word problems, which were not relevant for the research question. As outlined in the core manuscript, there were two parallel versions of each word problem with the same story – one routine word problem and one non-routine word problem (Table B). The latter followed an uncommon structure or included an unexpected question. For instance, some non-routine filler tasks did not require calculating at all because the answer was included in the text.

**Table B**

*16 filler tasks, each story was constructed both as routine and non-routine word problem.*

| Type of filler task | |
| --- | --- |
| routine | non-routine |
| Oma schenkt ihren 3 Enkelkindern eine Tüte.  *(Grandma gives a bag to her 3 grandchildren.)*  In der Tüte sind 18 Luftballons.  *(There are 18 balloons in the bag.)*  Diese werden gerecht zwischen den Enkelkindern aufgeteilt.  *(These are divided fairly between the grandchildren.)*  Wie viele Luftballons bekommt jedes Enkelkind?  *(How many balloons does each grandchild get?)* | Oma schenkt ihren 5 Enkelkindern 18 Tüten.  *(Grandma gives 18 bags to her 5 grandchildren.)*  In jeder Tüte sind 5 Luftballons.  *(There are 5 balloons in each bag.)*  Diese werden gerecht zwischen den Enkelkindern aufgeteilt.  *(These are divided fairly between the grandchildren.)*  Wie viele Tüten mit je 5 Luftballons bekommt jedes Kind?  *(How many bags of 5 balloons does each child get?)* |
| Lena hat 6 Stoffbahnen gekauft.  *(Lena bought 6 panels of fabric.)*  Jede davon ist 4 Meter lang.  *(Each of them is 4 meters long.)*  Daraus möchte Lena 2 Meter lange Stoffbahnen zuschneiden.  *(Lena would like to cut 2-meter-long panels of fabric out of them.)*  Wie viele kürzere Stoffbahnen kann sie daraus zuschneiden?  *(How many shorter panels of fabric can she cut out of them?)* | Lena hat 6 Stoffbahnen gekauft.  *(Lena bought 6 panels of fabric.)*  Jede davon ist 5 Meter lang.  *(Each of them is 5 meters long.)*  Daraus möchte Lena 2 Meter lange Stoffbahnen zuschneiden.  *(Lena would like to cut 2-meter-long panels of fabric out of them.)*  Wie viele kürzere Stoffbahnen kann sie daraus zuschneiden?  *(How many shorter panels of fabric can she cut out of them?)* |
| Anna hat 16 Stifte.  *(Anna has 16 pencils.)*  Lukas hat 58 Stifte.  (Lukas has 58 pencils.)  Anna möchte genauso viele Stifte haben wie Lukas.  *(Anna would like to have as many pencils as Lukas.)*  Wie viele Stifte muss Anna dazu kaufen? *(How many pens does Anna have to buy?)* | Anna hat 26 Stifte.  *(Anna has 26 pencils.)*  Lukas hat 48 Stifte.  *(Lukas has 48 pencils.)*  Anna kauft sich 42 neue Stifte.  *(Anna buys 42 new pens.)*  Wie viele Stifte hat Lukas?  *(How many pens does Lukas have?)* |
| Ein Mann zerschneidet ein Seil.  *(A man cuts a rope into pieces.)*  Anfangs ist es 15 Meter lang.  *(At first, it is 15 meters long.)*  Er schneidet jeweils nach 5 Metern ein Stück ab.  *(He cuts off a piece after each 5 meters.)*  Wie viele kurze Seile hat der Mann am Ende?  *(How many short ropes does the man have at the end?)* | Ein Mann pflanzt Bäume an einen 15 Meter langen Weg.  *(A man plants trees along a 15-meter-long path.)*  Den ersten Baum pflanzt er am Anfang, den letzten am Ende.  *(He plants the first tree at the beginning, the last one at the end.)*  Er pflanzt alle 3 Meter einen Baum entlang des Weges.  *(He plants a tree every 3 meters along the path.)*  Wie viele Bäume pflanzt er insgesamt? *(How many trees does he plant in total?)* |
| In einem Restaurant stehen Kerzen auf den Tischen.  *(In a restaurant, there are candles on the tables.)*  Es brennen bereits 42 Kerzen.  *(There are 42 candles already burning.)*  26 Kerzen sind noch nicht angezündet.  *(26 candles are not yet lit.)*  Wie viele Kerzen stehen insgesamt auf den Tischen?  *(How many candles in total are on the tables?)* | In einem Restaurant stehen 52 Kerzen auf den Tischen.  *(In a restaurant, there are 52 candles on the tables.)*  Es brennen bereits 21 Kerzen.  *(There are 21 candles already burning.)*  31 Kerzen sind noch nicht angezündet.  *(31 candles are not yet lit.)*  Wie viele Kerzen stehen insgesamt auf den Tischen?  *(How many candles in total are there on the tables?)* |
| Eine zylinderförmige Vase wird konstant mit Wasser befüllt.  *(A cylindrical vase is being consistently filled with water.)*  Die Vase ist 20 cm hoch.  *(The vase is 20 cm high.)*  Nach 10 Sekunden steht das Wasser 4 cm hoch.  *(After 10 seconds, the water level is at 4 cm.)*  Wie hoch steht das Wasser nach insgesamt 30 Sekunden?  *(How high is the water level after a total of 30 seconds?)* | Eine zylinderförmige Vase wird konstant mit Wasser befüllt.  *(A cylindrical vase is being consistently filled with water.)*  Die Vase ist 10 cm hoch.  *(The vase is 10 cm high.)*  Nach 10 Sekunden steht das Wasser 5 cm hoch.  *(After 10 seconds, the water level is at 5 cm.)*  Wie hoch steht das Wasser nach insgesamt 30 Sekunden?  *(How high is the water level after a total of 30 seconds?)* |
| Ein Mann möchte einen Weg mit Steinplatten verlegen.  *(A man wants to lay a path with flagstones.)*  Der Weg ist 12 Meter lang.  *(The path is 12 meters long.)*  Dafür besorgt er jeweils 2 Meter lange Steinplatten.  *(For this purpose, he procures 2-meter-long flagstones.)*  Wie viele von diesen Steinplatten braucht er für den Weg?  *(How many of these flagstones does he need for the path?)* | Ein Mann möchte einen Gartenstreifen mit Rollrasen bepflanzen.  *(A man wants to plant a strip of garden with turf.)*  Der Gartenstreifen ist 14 Meter lang.  *(The strip of garden is 14 meters long.)*  Dafür besorgt er jeweils 4 Meter lange Stücke Rollrasen.  *(For this purpose, he procures 4-meter-long pieces of sod.)*  Wie viele von diesen Stücken braucht er für den Gartenstreifen?  *(How many of these pieces does he need for the garden strip?)* |
| Die Soldaten werden mit Bussen zu ihrem Trainingsplatz gefahren.  *(The soldiers are driven to their training site by buses.)*  Heute nehmen 250 Soldaten am Training teil.  *(Today, 250 soldiers participate in the training.)*  Jeder Bus kann 50 Soldaten transportieren. *(Each bus can transport 50 soldiers.)*  Wie viele Busse werden heute benötigt? *(How many buses are needed today?)* | Die Soldaten werden mit Bussen zu ihrem Trainingsplatz gefahren.  *(The soldiers are driven to their training site by buses.)*  Heute nehmen 250 Soldaten am Training teil.  *(Today, 250 soldiers participate in the training.)*  Jeder Bus kann 20 Soldaten transportieren. *(Each bus can transport 20 soldiers.)*  Wie viele Busse werden heute benötigt? *(How many buses are needed today?)* |

**Supplementary Material C:**

**Assessment of cognitive covariates by neuropsychological tests**

After the main part of the study with 32 word problems and 16 filler tasks, a battery of neuropsychological tests was administered to the participants to ensure the samples’ cognitive functioning was representative. Namely, three types of creativity, basic mathematical competencies, and working memory capacity were tested.

**Procedure**

First, we assessed figural creativity with the LO subtest of the Berliner Intelligenzstrukturtest für Jugendliche: Begabungs- und Hochbegabungsdiagnostik (BIS-HB; Jäger et al., 2006). Participants were asked to draw as many logos as they could come up with for a bike store within three minutes. Each invented logo that was no repetition and that contained a bike was awarded one point. The results are evaluated by two raters, and the final score is the mean of the two raters' scores.

Second, verbal creativity was assessed with the two sentence combination tasks of Analyse des Schlussfolgernden und Kreativen Denkens (ASK; Schuler and Hell, 2005). In each task, four letters were given to the participants, who were asked to construct as many four-word sentences as possible within three minutes, while using the four letters as initial letters for the words in any order. Each sentence with meaningful content containing a subject, a predicate, and an object was awarded one point. ASK is measured in two rounds using different letter stimuli. The results are evaluated by two raters, and the final score is the mean of the two raters' scores.

Third, numerical creativity was tested with the DR1 subtest of the BIS-HB (Jäger et al., 2006). Participants were asked to find as many combinations of three numbers as possible within 90 seconds to reach a result of 60 by multiplying the first with the second number and adding the third number (__ * __ + __ = 60). Every mathematically correct equation was evaluated with one point. If one number was kept constant across several solutions or only the order of the two factors in the multiplication was reversed, the answers were counted as repetitions and only up to five repetitions of each kind were given a point. For all three creativity measures (figural, verbal, and numerical), the respective final score consisted of sum of the awarded points. The results are evaluated by two raters, and the final score is the mean of the two raters' scores.

Fourth, two speed calculation tests for addition and subtraction (Huber et al., 2013) were completed. Each of them consisted of 28 items with two two-digit numbers in mathematical notation without text (e.g., 22 + 19 in the speed addition test, or 64 − 47 in the speed subtraction test). Participants were asked to complete as many items correctly as possible within 1.5 minutes in each of the two tests. Basic mathematical competencies were evaluated separately for addition and subtraction and quantified by the number of correctly solved items in the corresponding speed calculation test.

Fifth, verbal working memory capacity was assessed with two letter span tasks, where the experimenter read out loud a random letter sequence, which had to be reproduced by the participant in the correct order directly afterwards. The first two sequences consisted of two letters, and the length increased continuously with two sequences per length, stopping whenever a participant could not reproduce any of the two sequences of the same length or at a maximum of nine letters. The test was conducted once as a forward reproduction (name letters in same order as the experimenter), and once as a backward reproduction (name letters in reversed order). The final score was the number of correctly reproduced letter spans.

Sixth, spatial working memory capacity was assessed via the Corsi Block-Tapping Test (Corsi, 1972). In each trial, the experimenter tapped on several of nine blocks places irregularly on a board. Participants had to reproduce the block sequence in the correct order and received feedback on accuracy. The first two sequences consisted of two blocks, but the sequence length increased over trials with three sequences per length. Similar to the letter span task, participants completed a forward and backward version. The test was stopped when a participant could not reproduce at least two of the three sequences of a length. The final score was number of correctly answered trials.

**Results**

The results of all neuropsychological tests described above can be found in Table C.

**Table C**

*Averaged results for the cognitive covariates assessed by neuropsychological tests (standard deviations in parenthesis).*

| Cognitive covariate | Test used for assessment | Mean score (SD) |
| --- | --- | --- |
| Figural creativity | LO subtest of BIS-HB | 4.48 (2.01) |
| Verbal creativity | ASK | 3.83 (1.71) |
| Numerical creativity | DR1 subtest of BIS-HB | 10.52 (3.59) |
| Basic mathematics | Speeded addition | 18.24 (5.44) |
| Basic mathematics | Speeded subtraction | 14.30 (5.40) |
| Verbal working memory capacity | Forward letter span | 8.62 (1.53) |
| Verbal working memory capacity | Backward letter span | 6.46 (1.70) |
| Figural working memory capacity | Forward Corsi score | 14.44 (2.33) |
| Figural working memory capacity | Backward Corsi score | 14.05 (2.93) |

**Supplementary Material D:**

**Static eye-tracking measures for solvable word problems**

The pattern of all static eye-tracking measures (FD, FC, RD, and RC) for the numbers (AOI) in correctly answered solvable word problems was similar. In all four variables, a significant interaction effect revealed that, as compared to the switch from addition to subtraction and from simple to complex, there was an overadditive effect when switching from simple addition to complex subtraction (see Tables D1, D2, D3, and D4). In other words, the borrow effect in subtraction is larger than the carry effect in addition, as can be seen in Figure D. Moreover, the baseline of all static eye-tracking measures varied between participants, as reflected by significant random intercepts in all four LMMs. Because of the significant interaction effect, lower-level effects (i.e., main effects operation and difficulty) were not tested but remained in the LMM. As compared to the reference category of simple addition problems, descriptively more attention was drawn to simple subtraction and complex addition problems. Note that the results are similar for all four static eye-tracking variables, because FD, FC, RD, and RC are not independent from one another.

**Table D1**

*This table displays the LMM results for fixation durations (FD; in milliseconds) on the numbers (AOI) within correctly answered solvable word problems. The parameter estimates refer to the LMM equation explained in the paragraph “Data analysis” of the “Method” section. Dummy-coding was used, with the fixed intercept reflecting the estimate for non-carry addition word problems and serving as reference category. Parameter estimates for other factor levels revealing significant fixed effects refer to differences between the reference category and the respective factor level (Add = addition, Sub = subtraction, NCNB = non-carry/non-borrow, CB = carry/borrow). For fixed effects (β), the F‑test statistic is displayed, whereas for random effects (S), the Likelihood-Ratio-Test (LRT) statistic is displayed. Estimates for random effects (i.e., intercept variabilities between participants P and word problems W) are standard deviations. The last column shows the p‑value corresponding to the F-test or to the LRT, with an asterisk for significant predictors of FD.*

| **Parameter** | **Meaning** | **Estimate** | **F‑test / LRT** | **p‑value** |
| --- | --- | --- | --- | --- |
| *β*_0_ | Fixed: intercept_NCNB-Add_ | 2694.95 | - | - |
| *S*_0_*_Pi_* | Random: participant | 1103.80 | 128.71 | < .001* |
| *β_1_* | Fixed: difficulty_CB_ | 658.76 | - | - |
| *β_2_* | Fixed: operation_Sub_ | 309.50 | - | - |
| *β_3_* | Fixed: interaction_CB-Sub_ | 665.29 | 6.26 | .013* |

**Table D2**

*This table displays the LMM results for fixation counts (FC) on the numbers (AOI) within correctly answered solvable word problems (for further explanations, see description of Table D1).*

| **Parameter** | **Meaning** | **Estimate** | **F‑test / LRT** | **p‑value** |
| --- | --- | --- | --- | --- |
| *β*_0_ | Fixed: intercept_NCNB-Add_ | 7.75 | - | - |
| *S*_0_*_Pi_* | Random: participant | 2.66 | 137.55 | < .001* |
| *β_1_* | Fixed: difficulty_CB_ | 0.24 | - | - |
| *β_2_* | Fixed: operation_Sub_ | 0.38 | - | - |
| *β_3_* | Fixed: interaction_CB-Sub_ | 1.52 | 5.98 | .015* |

**Table D3**

*This table displays the LMM results for regression durations (RD; in milliseconds) on the numbers (AOI) within correctly answered solvable word problems (for further explanations, see description of Table D1).*

| **Parameter** | **Meaning** | **Estimate** | **F‑test / LRT** | **p‑value** |
| --- | --- | --- | --- | --- |
| *β*_0_ | Fixed: intercept_NCNB-Add_ | 2216.91 | - | - |
| *S*_0_*_Pi_* | Random: participant | 1058.98 | 116.15 | < .001* |
| *β_1_* | Fixed: difficulty_CB_ | 594.25 | - | - |
| *β_2_* | Fixed: operation_Sub_ | 284.48 | - | - |
| *β_3_* | Fixed: interaction_CB-Sub_ | 728.13 | 7.49 | .006* |

**Table D4**

*This table displays the LMM results for regression counts (RC) on the numbers (AOI) within correctly answered solvable word problems (for further explanations, see description of Table D1).*

| **Parameter** | **Meaning** | **Estimate** | **F‑test / LRT** | **p‑value** |
| --- | --- | --- | --- | --- |
| *β*_0_ | Fixed: intercept_NCNB-Add_ | 5.67 | - | - |
| *S*_0_*_Pi_* | Random: participant | 2.55 | 133.31 | < .001* |
| *β_1_* | Fixed: difficulty_CB_ | 0.25 | - | - |
| *β_2_* | Fixed: operation_Sub_ | 0.44 | - | - |
| *β_3_* | Fixed: interaction_CB-Sub_ | 1.48 | 5.98 | .015* |

**Figure D**

*This figure illustrates (A) FD in milliseconds, (B) FC, (C) RD in milliseconds, and (D) RC on the numbers (AOI) in solvable word problems. The mean is plotted for each operation (addition vs. subtraction) depending on difficulty (blue: non-carry/non-borrow; red: carry/borrow) with error bars indicating plus/minus one standard error.*

*
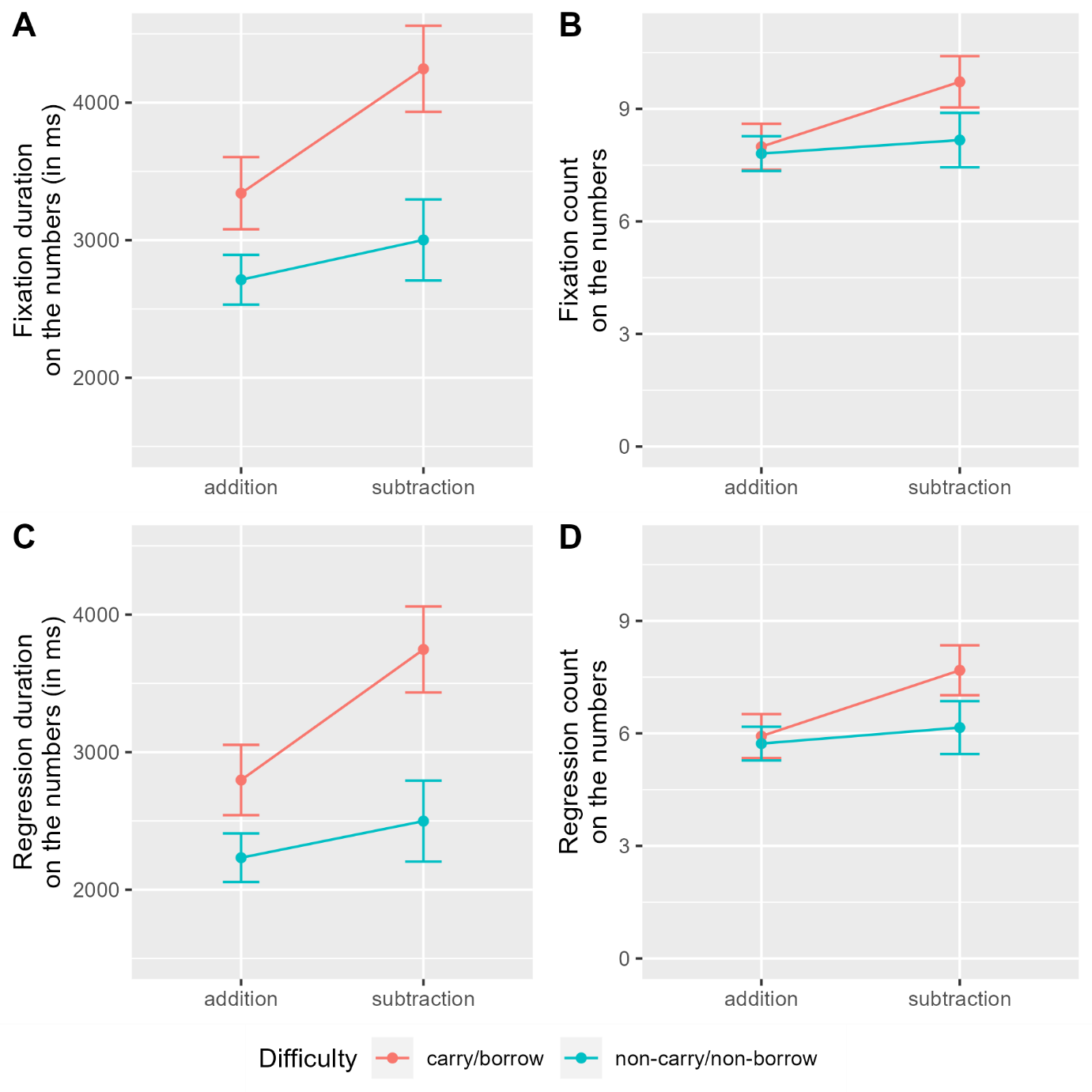
*

**Supplementary Material E:**

**Dynamic eye-tracking measures for solvable word problems**

The patterns of the dynamic eye-tracking measures (NN, TT, and TN) in correctly answered solvable word problems differed from one another and from the patterns of the static eye-tracking measures (FD, FC, RD, and RC). For NN transitions, no significant interaction between operation and difficulty was detected. However, a significant effect of difficulty was found, such that more NN transitions were observed in carry/borrow than in non-carry/non-borrow word problems (see Table E1). For TT transitions, a significant interaction between operation and difficulty revealed that, as compared to the switch from addition to subtraction and from simple to complex, there was an underadditive effect when switching from simple addition to complex subtraction (see Table E2). This crossover interaction is illustrated in Figure E (Panel B). Because of the significant interaction effect, lower-level effects (i.e., main effects operation and difficulty) were not tested but remained in the LMM. As compared to the reference category of simple addition problems, descriptively more transitions were made in simple subtraction and complex addition problems. For TN transitions, no significant interaction between operation and difficulty was detected. However, a significant effect of operation was found, such that more NN transitions were observed in subtraction than in addition word problems (see Table E3). In all three dynamic eye-tracking variables, the baseline varied between participants, as reflected by significant random intercepts in all three LMMs. Transitions made in solvable word problems are illustrated in Figure E.

**Table E1**

*This table displays the LMM results for NN transition counts within correctly answered solvable word problems (for further explanations, see description of Table D1).*

| **Parameter** | **Meaning** | **Estimate** | **F‑test / LRT** | **p‑value** |
| --- | --- | --- | --- | --- |
| *β*_0_ | Fixed: intercept_NCNB-Add_ | 2.93 | - | - |
| *S*_0_*_Pi_* | Random: participant | 1.94 | 120.21 | < .001* |
| *β_1_* | Fixed: difficulty_CB_ | 1.07 | 19.76 | < .001* |
| *β_2_* | Fixed: operation_Sub_ | 0.57 | 5.68 | .017* |
| *β_3_* | Fixed: interaction_CB-Sub_ | - | 2.68 | .102 |

**Table E2**

*This table displays the LMM results for TT transition counts within correctly answered solvable word problems (for further explanations, see description of Table D1).*

| **Parameter** | **Meaning** | **Estimate** | **F‑test / LRT** | **p‑value** |
| --- | --- | --- | --- | --- |
| *β*_0_ | Fixed: intercept_NCNB-Add_ | 24.30 | - | - |
| *S*_0_*_Pi_* | Random: participant | 6.08 | 188.24 | < .001* |
| *β_1_* | Fixed: difficulty_CB_ | 1.28 | - | - |
| *β_2_* | Fixed: operation_Sub_ | 5.19 | - | - |
| *β_3_* | Fixed: interaction_CB-Sub_ | -2.90 | 5.36 | .021* |

**Table E3**

*This table displays the LMM results for TN transition counts within correctly answered solvable word problems (for further explanations, see description of Table D1).*

| **Parameter** | **Meaning** | **Estimate** | **F‑test / LRT** | **p‑value** |
| --- | --- | --- | --- | --- |
| *β*_0_ | Fixed: intercept_NCNB-Add_ | 8.15 | - | - |
| *S*_0_*_Pi_* | Random: participant | 1.70 | 52.08 | < .001* |
| *β_1_* | Fixed: difficulty_CB_ | - | 0.43 | .514 |
| *β_2_* | Fixed: operation_Sub_ | 1.20 | 16.55 | < .001* |
| *β_3_* | Fixed: interaction_CB-Sub_ | - | 2.86 | .091 |

**Figure E**

*This figure illustrates (A) NN transitions, (B) TT transitions, and (C) TN transitions in solvable word problems. The mean is plotted for each operation (addition vs. subtraction) depending on difficulty (blue: non-carry/non-borrow; red: carry/borrow) with error bars indicating plus/minus one standard error.*

**
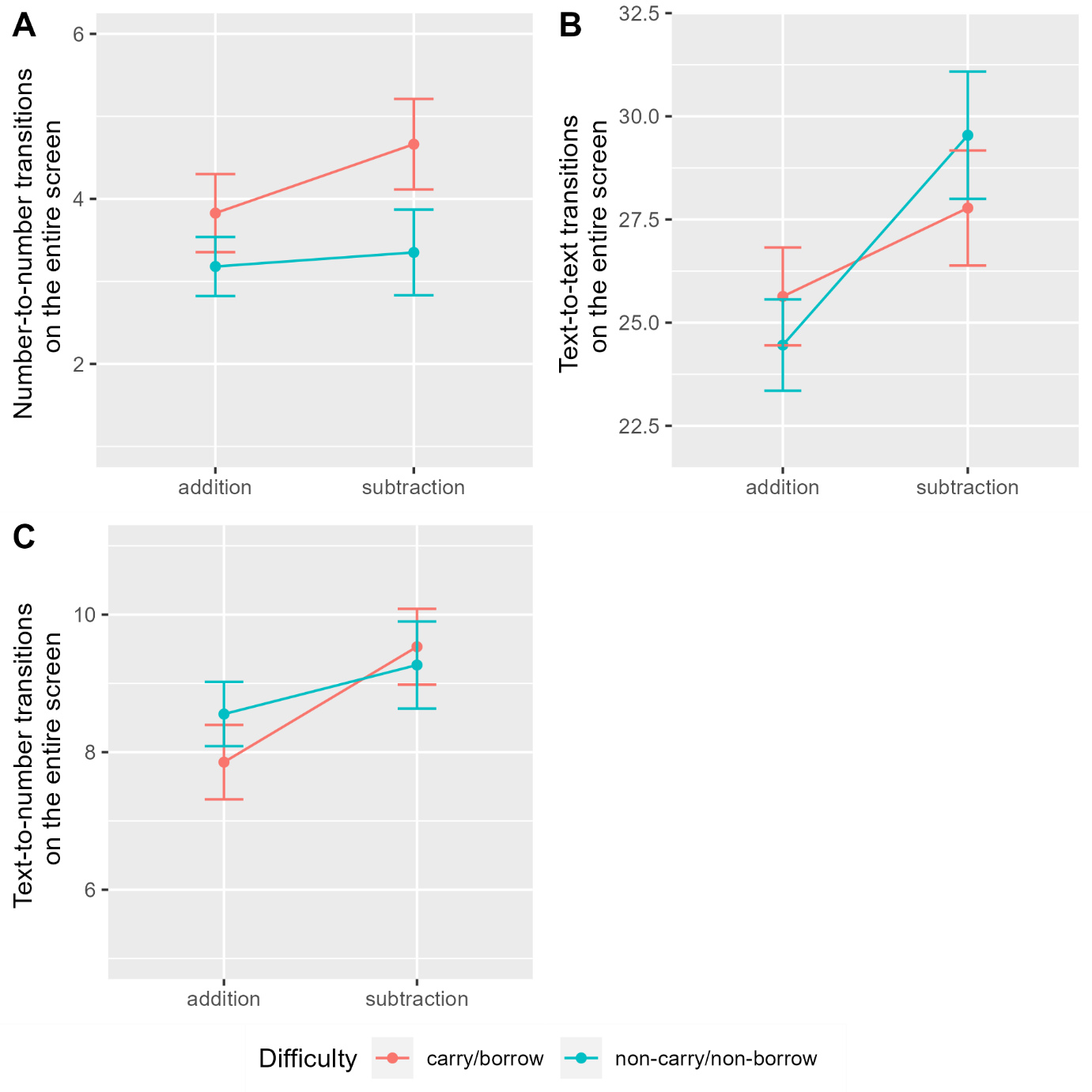
**

**Supplementary Material F:**

**Performance measures for solvable word problems**

The performance measures (RT and ER) in solvable word problems revealed a similar pattern, both not showing a significant interaction of operation and difficulty but main effects of both factors. More precisely, responses until a correct answer was given were slower and ER were higher in subtraction than in addition and in carry/borrow than in non-carry/non-borrow word problems (see Tables F1, and F2). Moreover, the baseline of RT and ER varied between participants, as reflected by significant random intercepts in both LMMs. RT for correctly answered solvable word problems and ER for solvable word problems are illustrated in Figure F.

**Table F1**

*This table displays the LMM results for RT (in seconds) within correctly answered solvable word problems (for further explanations, see description of Table D1).*

| **Parameter** | **Meaning** | **Estimate** | **F‑test / LRT** | **p‑value** |
| --- | --- | --- | --- | --- |
| *β*_0_ | Fixed: intercept_NCNB-Add_ | 11.34 | - | - |
| *S*_0_*_Pi_* | Random: participant | 2.47 | 297.38 | < .001* |
| *β_1_* | Fixed: difficulty_CB_ | 1.38 | 46.49 | < .001* |
| *β_2_* | Fixed: operation_Sub_ | 1.96 | 92.79 | < .001* |
| *β_3_* | Fixed: interaction_CB-Sub_ | - | 0.13 | .720 |

**Table F2**

*This table displays the LMM results for ER within solvable word problems (for further explanations, see description of Table D1).*

| **Parameter** | **Meaning** | **Estimate** | **F‑test / LRT** | **p‑value** |
| --- | --- | --- | --- | --- |
| *β*_0_ | Fixed: intercept_NCNB-Add_ | 0.06 | - | - |
| *S*_0_*_Pi_* | Random: participant | 0.06 | 5.24 | .022* |
| *β_1_* | Fixed: difficulty_CB_ | 0.07 | 11.43 | < .001* |
| *β_2_* | Fixed: operation_Sub_ | 0.05 | 5.17 | .023* |
| *β_3_* | Fixed: interaction_CB-Sub_ | - | 0.18 | .667 |

**Figure F**

*This figure illustrates (A) RT in seconds for correctly answered solvable word problems, and (B) ER for all solvable word problems. The mean is plotted for each operation (addition vs. subtraction) depending on difficulty (blue: non-carry/non-borrow; red: carry/borrow) with error bars indicating plus/minus one standard error.*


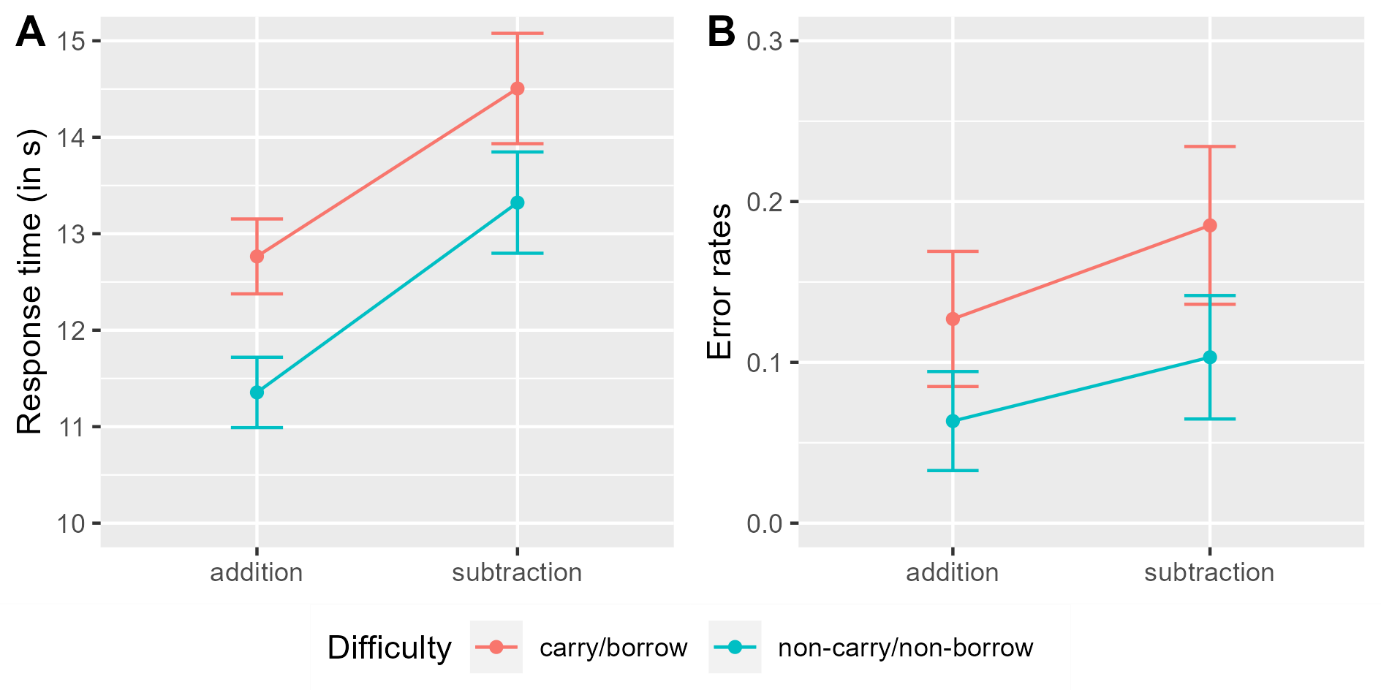


**Supplementary Material G:**

**Static eye-tracking measures for non-solvable word problems – entire screen**

Apart from investigating fixations on the numbers (AOI) in correctly answered non-solvable word problems, we also had a look at fixations on the entire screen (i.e., not only numbers, but also text or empty areas without numbers or text). In both FD and FC, a significant interaction effect revealed that, as compared to the switch from addition to subtraction and from simple to complex, there was an overadditive effect when switching from simple addition to complex subtraction (see Tables G1, and G2). This crossover interaction is illustrated in Figure G. Moreover, the baseline of both static eye-tracking measures varied between participants, as reflected by significant random intercepts in both LMMs. Because of the significant interaction effect, lower-level effects (i.e., main effects operation and difficulty) were not tested but remained in the LMM. As compared to the reference category of simple addition problems, descriptively less attention was drawn to simple subtraction and complex addition problems. Note that the results are similar for FD and FC, because they are not independent from one another.

**Table G1**

*This table displays the LMM results for FD (in milliseconds) on the entire screen within correctly answered non-solvable word problems (for further explanations, see description of Table D1).*

| **Parameter** | **Meaning** | **Estimate** | **F‑test / LRT** | **p‑value** |
| --- | --- | --- | --- | --- |
| *β*_0_ | Fixed: intercept_NCNB-Add_ | 11100.03 | - | - |
| *S*_0_*_Pi_* | Random: participant | 2206.53 | 104.99 | < .001* |
| *β_1_* | Fixed: difficulty_CB_ | -1798.92 | - | - |
| *β_2_* | Fixed: operation_Sub_ | -1744.70 | - | - |
| *β_3_* | Fixed: interaction_CB-Sub_ | 4069.33 | 51.54 | < .001* |

**Table G2**

*This table displays the LMM results for FC on the entire screen within correctly answered non-solvable word problems (for further explanations, see description of Table D1).*

| **Parameter** | **Meaning** | **Estimate** | **F‑test / LRT** | **p‑value** |
| --- | --- | --- | --- | --- |
| *β*_0_ | Fixed: intercept_NCNB-Add_ | 46.95 | < .001* | < .001* |
| *S*_0_*_Pi_* | Random: participant | 9.45 | 92.18 | < .001* |
| *β_1_* | Fixed: difficulty_CB_ | -7.26 | - | - |
| *β_2_* | Fixed: operation_Sub_ | -7.16 | - | - |
| *β_3_* | Fixed: interaction_CB-Sub_ | 18.09 | 54.89 | < .001* |

**Figure G**

*This figure illustrates (A) FD in milliseconds, and (B) FC on the entire screen in non-solvable word problems. The mean is plotted for each operation (addition vs. subtraction) depending on difficulty (blue: non-carry/non-borrow; red: carry/borrow) with error bars indicating plus/minus one standard error.*


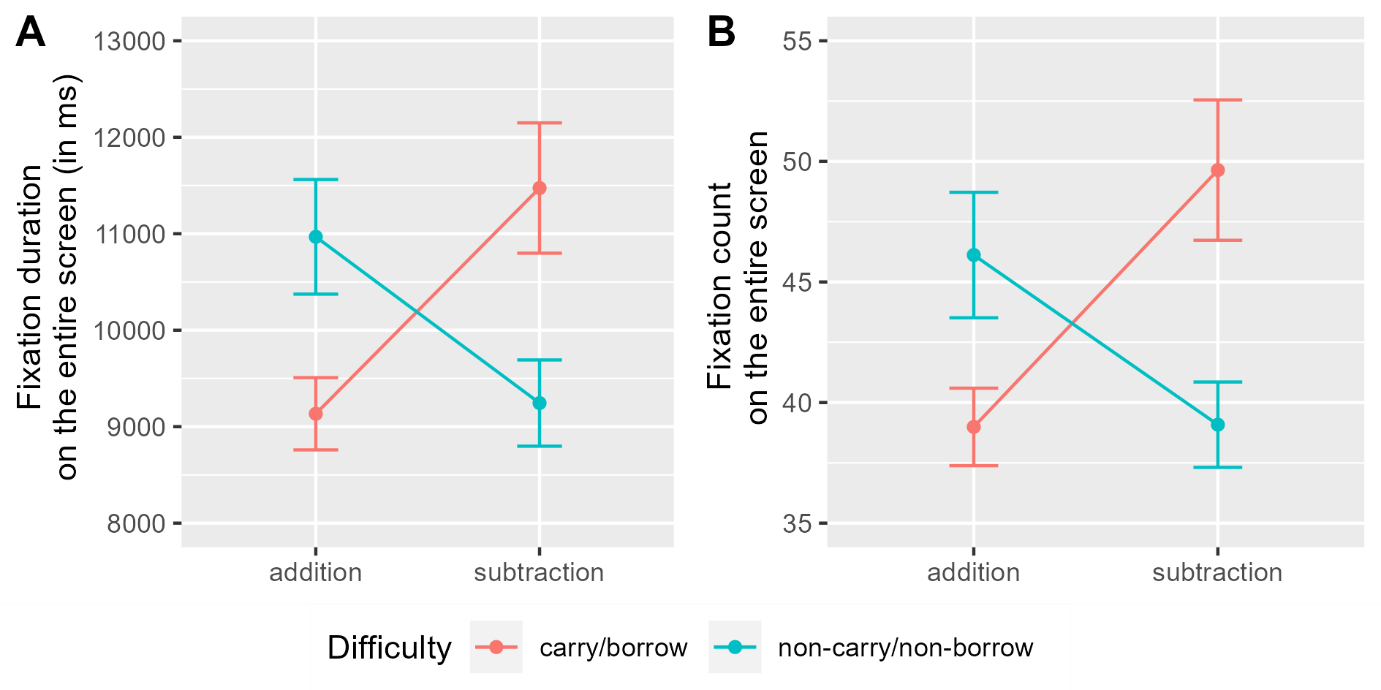


**Supplementary Material H:**

**Accuracies and error rates (ER) for each word problem story**

As described in the core manuscript, accuracies and ER were calculated across each pair of parallel word problems using the same story. This way, we were able to find out that the two non-solvable word problems built on the “Cinema” story were misunderstood and incorrectly answered by the majority of all participants (see Table H1). As expected, accuracies were high and ER were low for all word problem stories in the solvable versions (see Table H2).

**Table H1**

*This table displays accuracies and ER averaged over each pair of parallel non-solvable word problems differing in operation (addition vs. subtraction) but based on the same story. The asterisk indicated that the minimum of 33% accuracy or the maximum of 67% ER was not reached for one pair of parallel non-solvable word problems built on the same story.*

| **Word problem story** | **Accuracy** | **ER** |
| --- | --- | --- |
| Tennis | 0.9206 | 0.0794 |
| Marbles | 0.8492 | 0.1508 |
| Museum | 0.5635 | 0.4365 |
| Party | 0.3651 | 0.6349 |
| Gardening | 0.8810 | 0.1191 |
| Fruits | 0.9365 | 0.0635 |
| Cinema | 0.0714* | 0.9286* |
| Busses | 0.6111 | 0.3889 |

**Table H2**

*This table displays accuracies and ER averaged over each pair of parallel solvable word problems differing in operation (addition vs. subtraction) but based on the same story.*

| **Word problem story** | **Accuracy** | **ER** |
| --- | --- | --- |
| Tennis | 0.9286 | 0. 0714 |
| Marbles | 0.7778 | 0.2222 |
| Museum | 0.9206 | 0.0794 |
| Party | 0.8254 | 0.1746 |
| Gardening | 0.8730 | 0.1270 |
| Fruits | 0.8571 | 0.1429 |
| Cinema | 0.9127 | 0.0873 |
| Busses | 0.9841 | 0.0159 |

**Supplementary Material I:**

**Data points for each participant in each word problem**

This Supplementary Material includes nine figures, each for one dependent variable (I1: FD, I2: FC, I3: RD, I4: RC, I5: NN, I6: TT, I7: TN, I8: RT, and I9: ER). Each of these figures contains four plots depending on solvability and operation (one for solvable addition, solvable subtraction, non-solvable addition, and non-solvable subtraction word problems each).

**Figure I1**

*This figure illustrates FD in milliseconds on the numbers (AOI) in every single word problem. The word problems are split into four violin plots depending on solvability and operation, while difficulty is color-coded. Each of the jittered data points reflects one experimental trial, so that there is one data point for each of the 63 participants and for each of the seven stories in each of the four plots (story “Cinema” was excluded, see Supplementary Material H)*


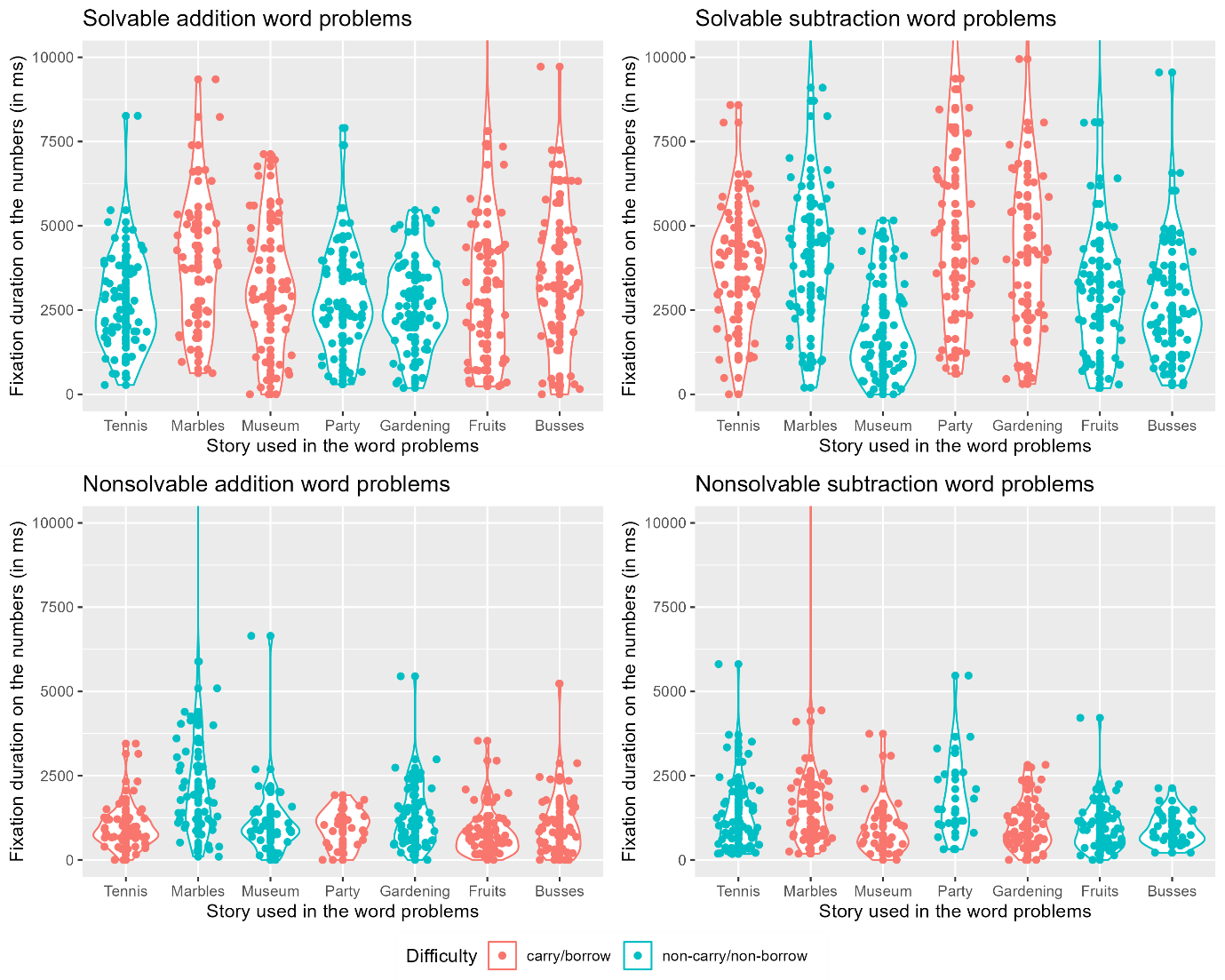


**Figure I2**

*This figure illustrates FC on the numbers (AOI) in every single word problem (for further explanations, see description of Table F1).*


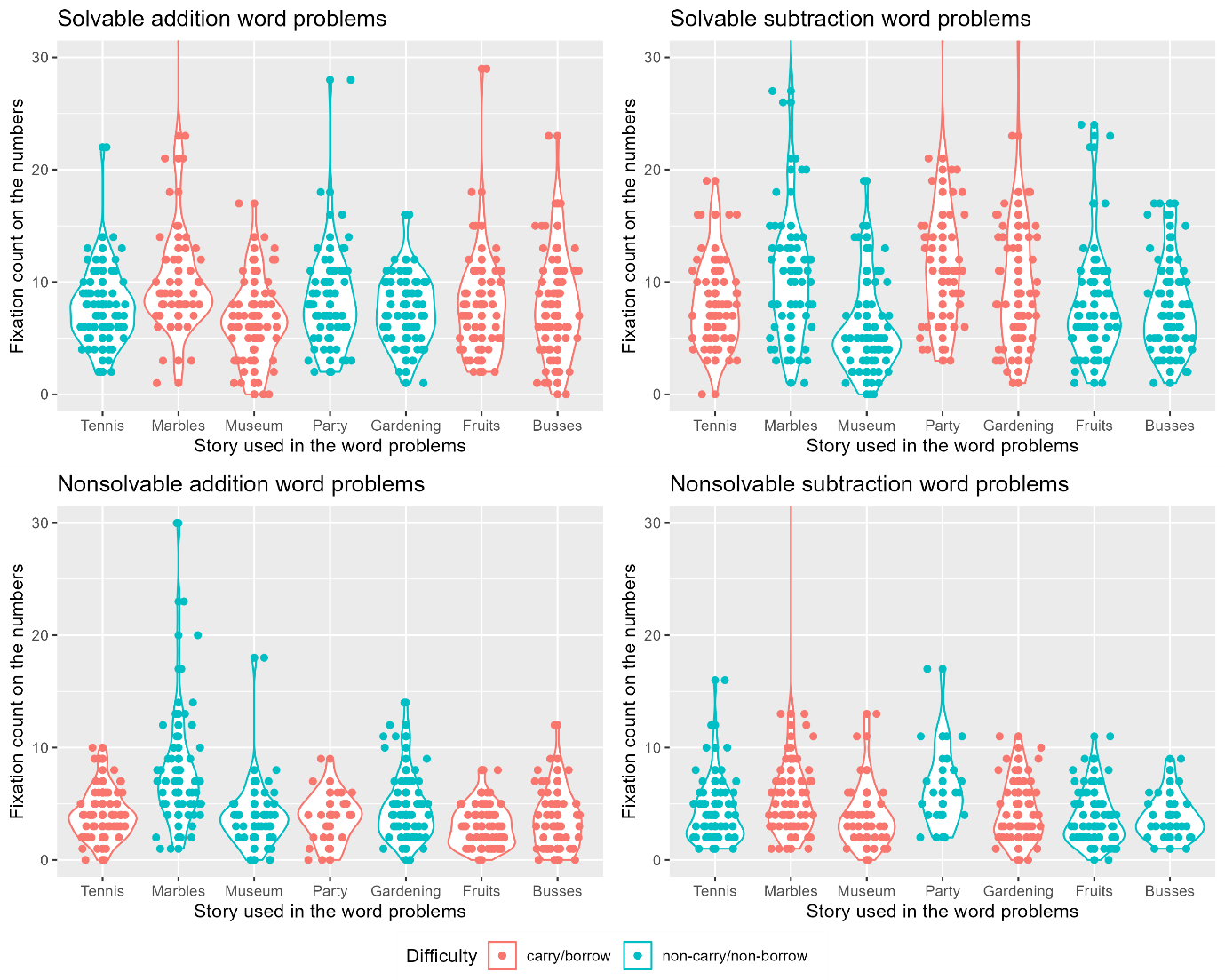


**Figure I3**

*This figure illustrates RD in milliseconds on the numbers (AOI) in every single word problem (for further explanations, see description of Table F1).*


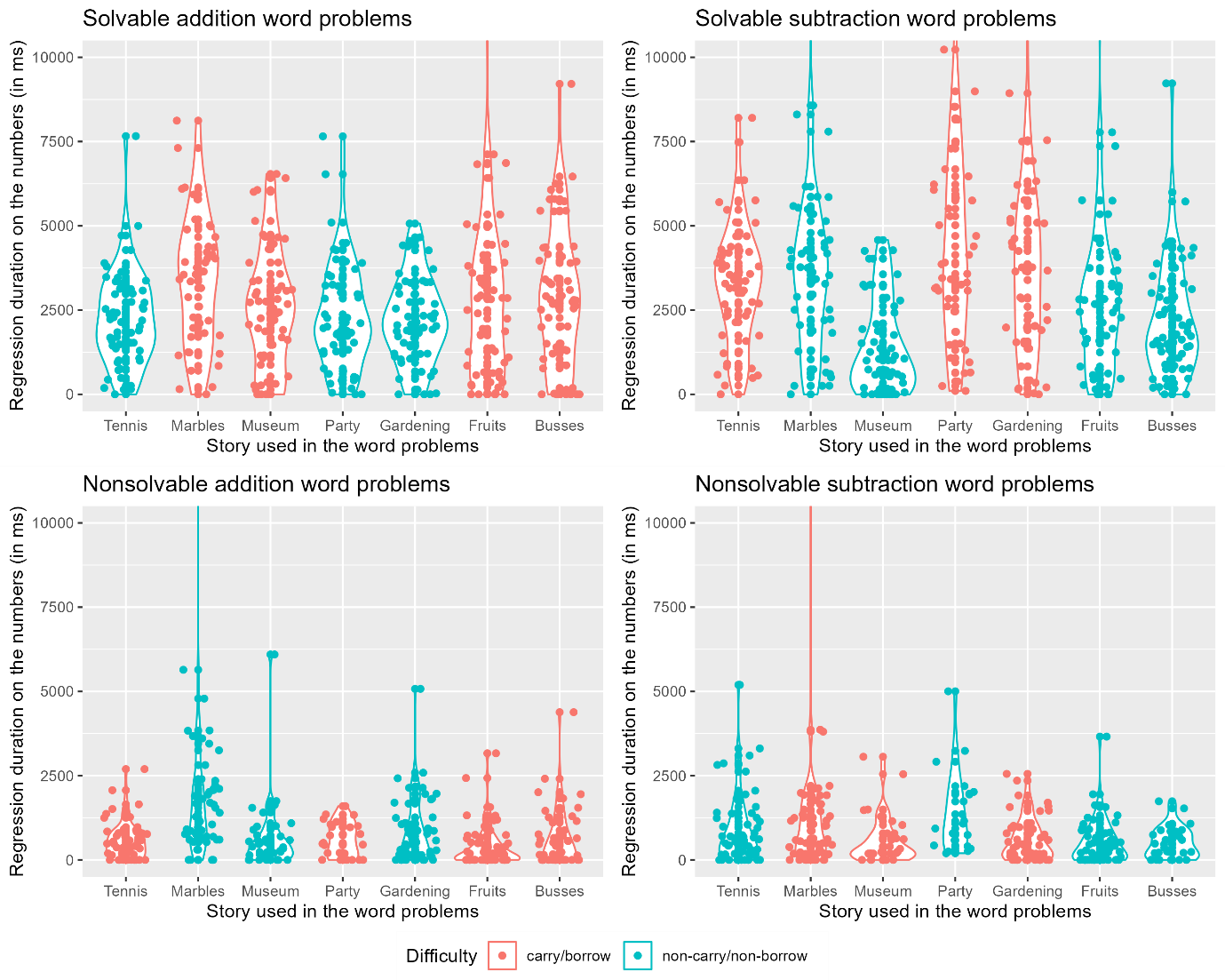


**Figure I4**

*This figure illustrates RC on the numbers (AOI) in every single word problem (for further explanations, see description of Table F1).*


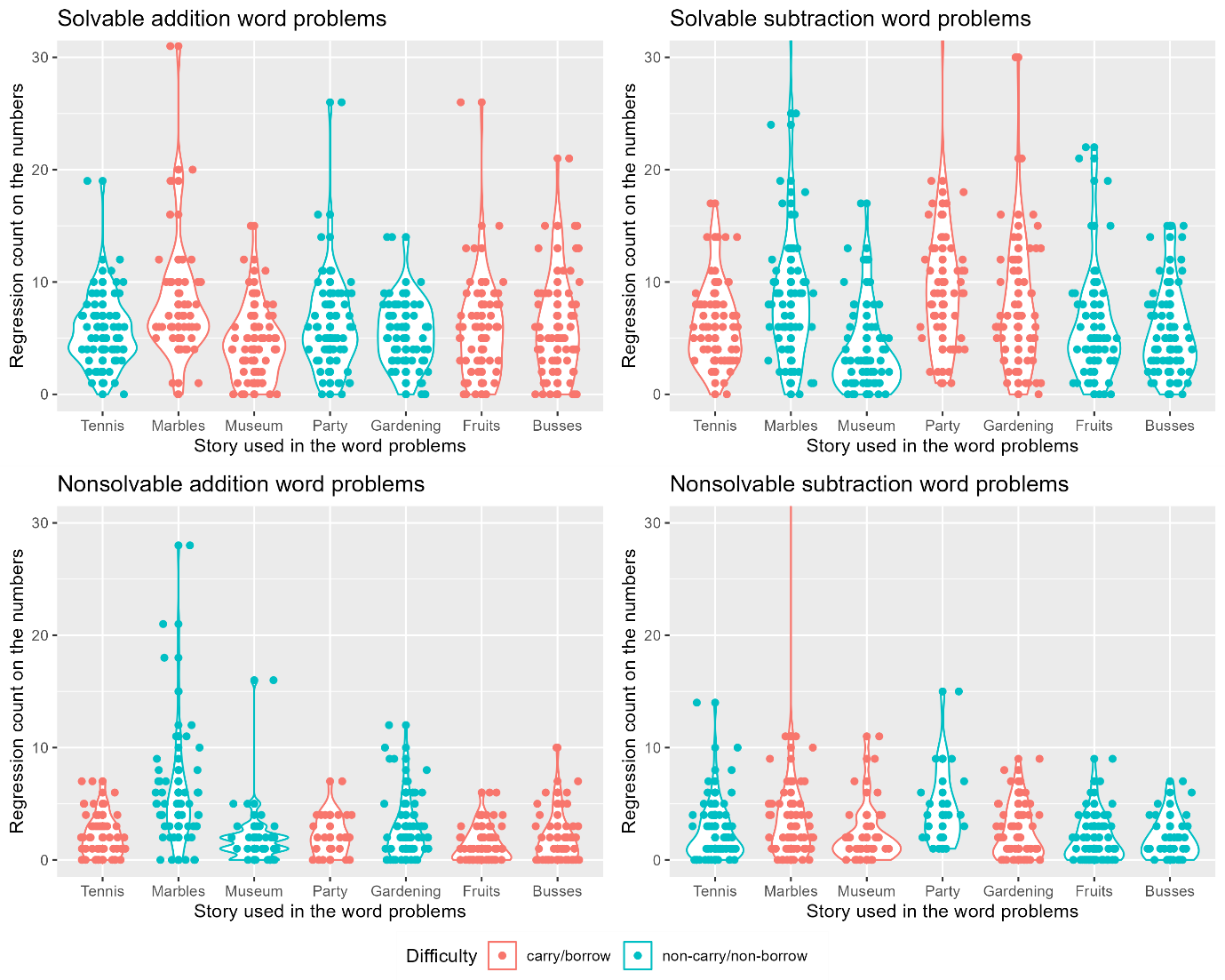


**Figure I5**

*This figure illustrates NN transitions in every single word problem (for further explanations, see description of Table F1).*


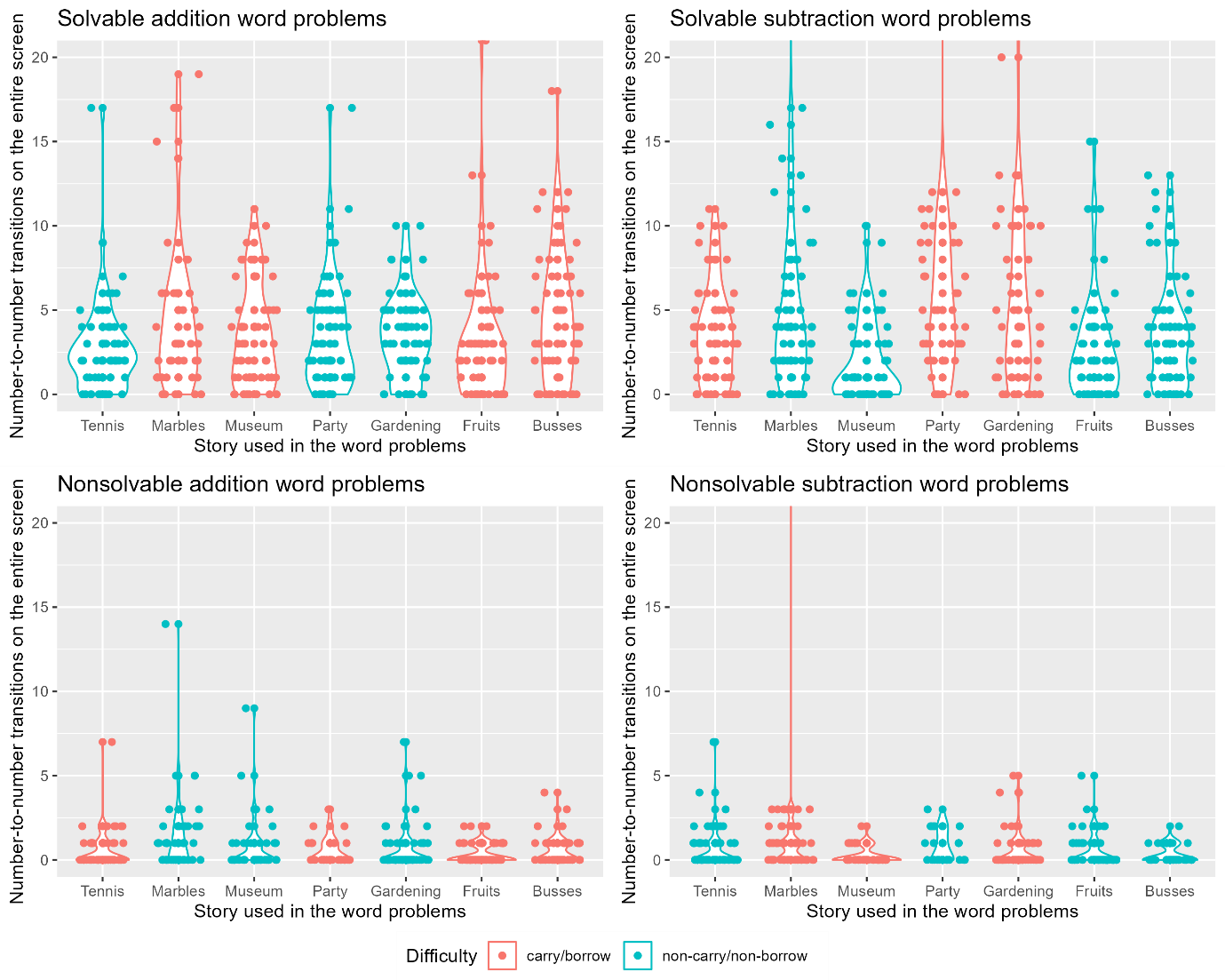


**Figure I6**

*This figure illustrates TT transitions in every single word problem (for further explanations, see description of Table F1).*


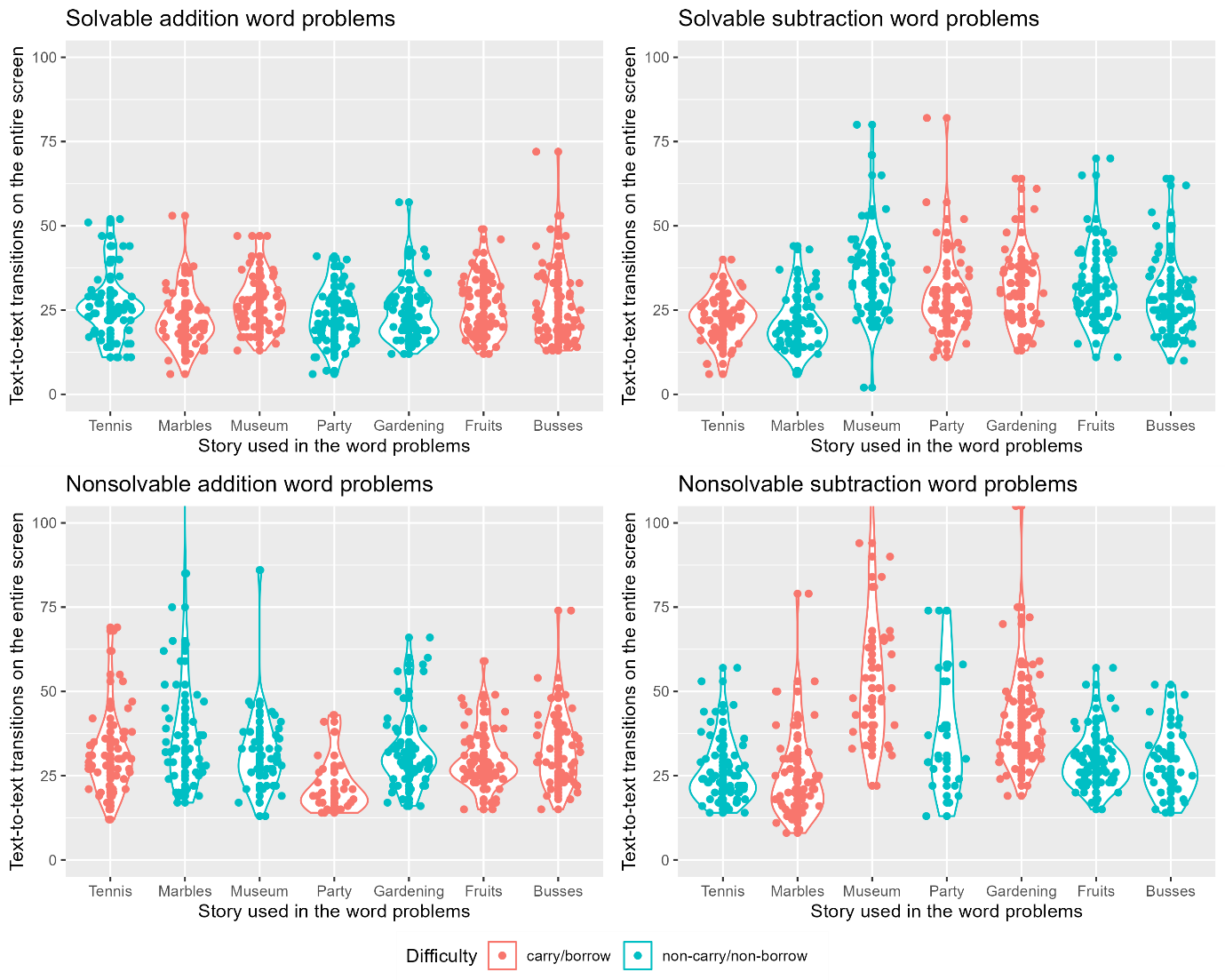


**Figure I7**

*This figure illustrates TN transitions in every single word problem (for further explanations, see description of Table F1).*


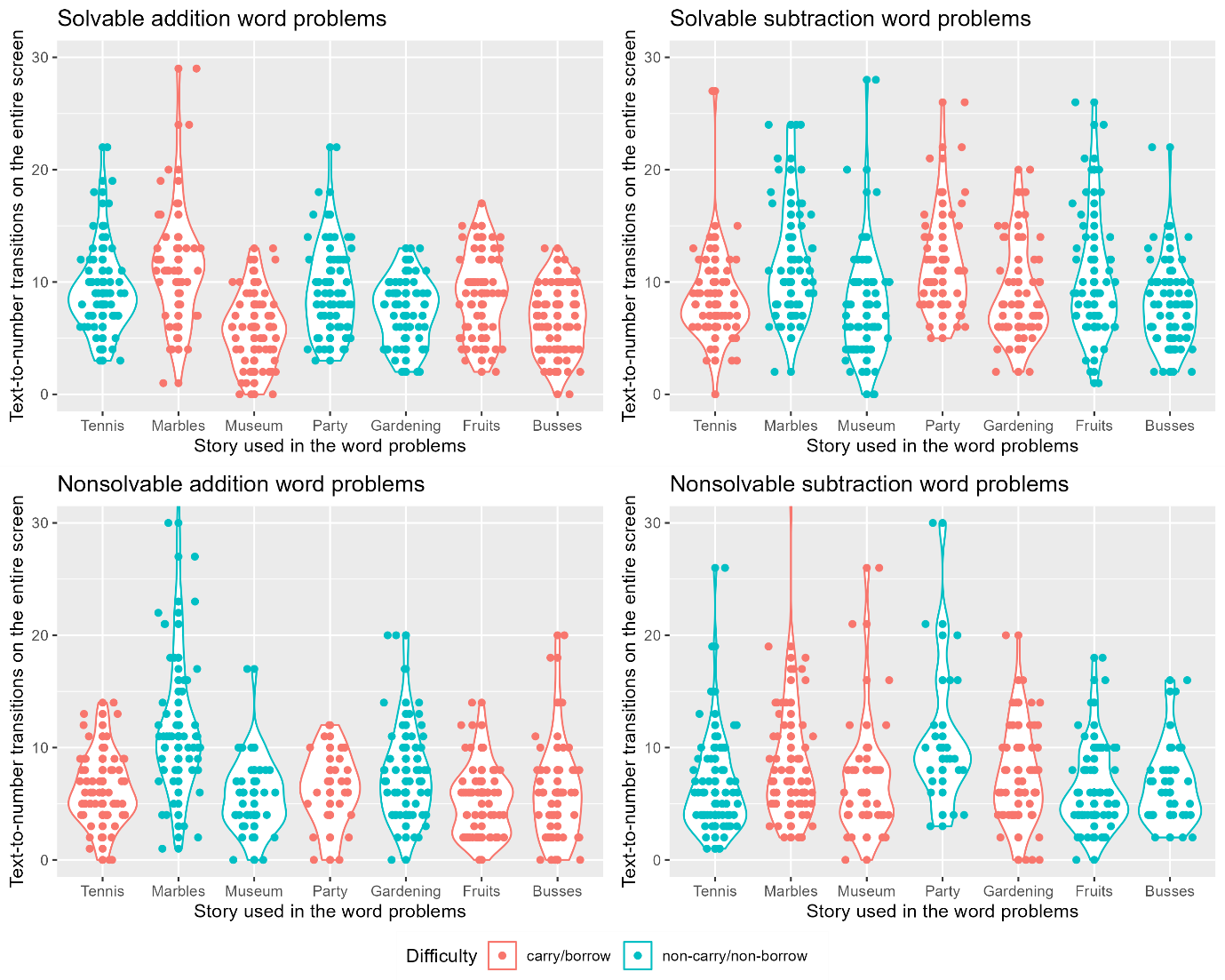


**Figure I8**

*This figure illustrates RT in seconds for every single word problem (for further explanations, see description of Table F1).*


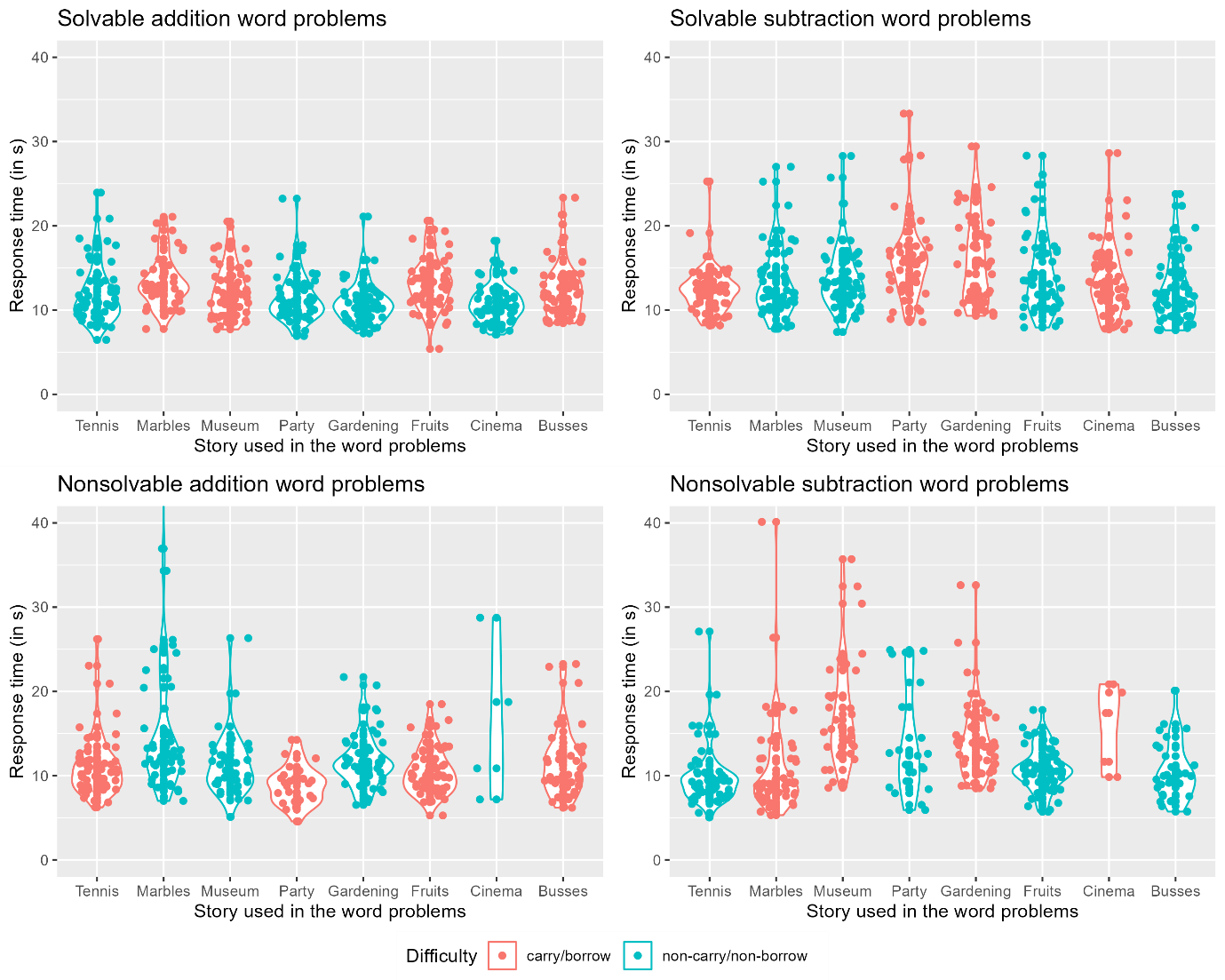


**Figure I9**

*This figure illustrates ER in every single word problem (for further explanations, see description of Table F1).*


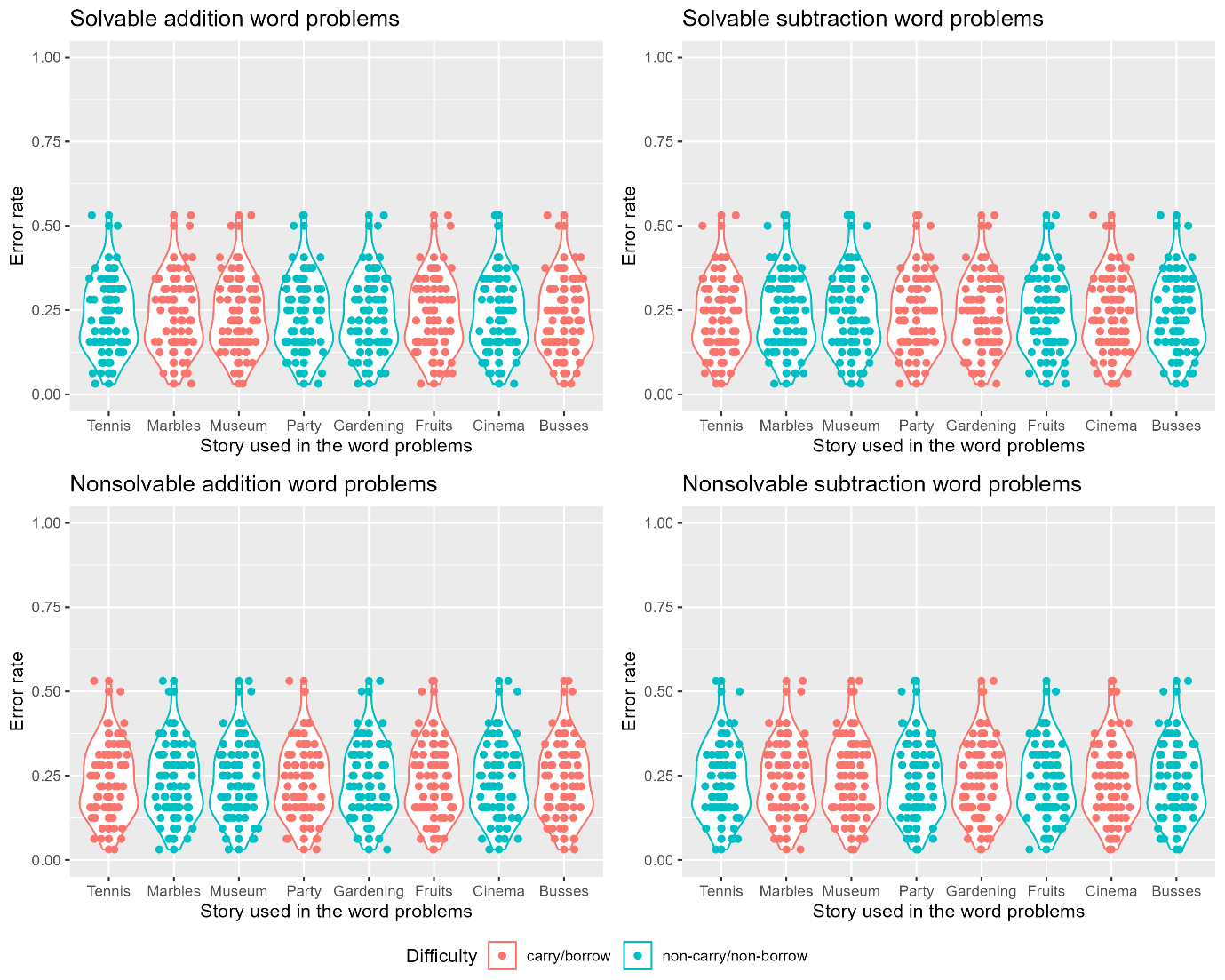

Supplement: Supplementary file 1 — Supplementary file1 (DOCX 3384 KB) [file 426_2024_2069_MOESM1_ESM.docx]
